# Effectiveness of a Worksite Mindfulness-Related Multi-Component Health Promotion Intervention on Work Engagement and Mental Health: Results of a Randomized Controlled Trial

**DOI:** 10.1371/journal.pone.0084118

**Published:** 2014-01-28

**Authors:** Jantien van Berkel, Cécile R. L. Boot, Karin I. Proper, Paulien M. Bongers, Allard J. van der Beek

**Affiliations:** 1 Department of Public and Occupational Health - EMGO Institute for Health and Care Research, VU University Medical Center, Amsterdam, the Netherlands; Body@Work, Research Center on Physical Activity, Work and Health, TNO-VU University Medical Center, Amsterdam, the Netherlands; 3 Department of Work and Employment, TNO Quality of Life, Hoofddorp, the Netherlands; Carnegie Mellon University, United States of America

## Abstract

**Objectives:**

The aim of the present study was to evaluate the effectiveness of a worksite mindfulness-related multi-component health promotion intervention on work engagement, mental health, need for recovery and mindfulness.

**Methods:**

In a randomized controlled trial design, 257 workers of two research institutes participated. The intervention group (n = 129) received a targeted mindfulness-related training, followed by e-coaching. The total duration of the intervention was 6 months. Data on work engagement, mental health, need for recovery and mindfulness were collected using questionnaires at baseline and after 6 and 12 months follow-up. Effects were analyzed using linear mixed effect models.

**Results:**

There were no significant differences in work engagement, mental health, need for recovery and mindfulness between the intervention and control group after either 6- or 12-months follow-up. Additional analyses in mindfulness-related training compliance subgroups (high and low compliance versus the control group as a reference) and subgroups based on baseline work engagement scores showed no significant differences either.

**Conclusions:**

This study did not show an effect of this worksite mindfulness-related multi-component health promotion intervention on work engagement, mental health, need for recovery and mindfulness after 6 and 12 months.

**Trial registration:**

Netherlands Trial Register NTR2199

## Introduction

Over the last decades, increasing automation and globalization have led to a change in daily working life in most sectors in the Western countries. Due to the associated increase in work pressure, competition, work pace, and job instability, work has become more mentally and emotionally demanding [1]–[3] This increase in mental and emotional demands may impair mental health. The consequences of impaired mental health at the workplace are serious, not only for the individual, but also for the organisation and society as a whole. Namely, impaired mental health is the second most frequent cause of absenteeism from work in Europe after musculoskeletal disorders [3], [4]. Globally, it is one of the leading causes for work disability [3].

Mental health is not merely the absence of disorders but has been defined by the WHO as “a state of well being in which every individual realizes his or her own potential, can cope with the normal stresses of life, can work productively and fruitfully, and is able to make a contribution to her or his community” [5]. A work-related indicator of subjective mental well-being is work engagement [6], [7]. Work engagement is a “positive, fulfilling, work-related state of mind that is characterized by vigor, dedication and absorption” [8], [9]. Work engagement has been shown to be negatively associated with burnout, depression, distress and psychosomatic complaints [9], [10] Furthermore, it can be seen as a predictor of long-term general well-being [11] and as a relevant workplace health promotion measure [12]. In addition, it is positively related to job performance [13] and has shown to be a (negative) predictor of sickness absenteeism [10] and a (positive) predictor for financial returns [14]. In summary, increasing work engagement is potentially beneficial for individual workers as well as their employers and society as a whole.

For this study, we developed a worksite intervention aimed at improving work engagement, called the ‘Mindful ‘Vitality In Practice’(VIP) intervention [15]. The main element of this intervention was a mindfulness-related training. According to Brown & Ryan, mindfulness can be described as “an open, undivided observation of what is occurring both internally and externally” [16] (p823). We expected to find an effect of the mindfulness-related intervention on work engagement, because it was hypothesized in the literature that increasing mindfulness would be an effective cognitive activity to increase work engagement [6], [7]. The working mechanism for increasing work engagement is that by becoming aware of thoughts, emotions and bodily sensations, and accepting them in a non-judging way, personal resources can be built. Personal resources are positive self-evaluations that are linked to resiliency and refer to individuals’ sense of their ability to cope with their environment successfully [8]. Examples of personal resources for work engagement are organizational-based self-esteem, self-efficacy, and optimism [8].

Positive effects of mindfulness-related training have been reported widely in reviews for treatment of mental disorders such as depression [16], [17]. It is also considered an effective strategy to improve general subjective well-being [16]. In addition, small scale research demonstrated beneficial effects for healthy working adults [18], which indicates that mindfulness-related training might not only be beneficial for treatment or secondary prevention objectives, but also for workplace health promotion objectives.

Other elements of the Mindful VIP intervention were e-coaching, fruit, lunch walking routes, and a buddy system. These elements aimed to increase resources of work engagement, such as self efficacy (through for example positive reinforcement by the e-coach) and social support (by forming pairs to discuss homework exercises or go lunch walking) [15].

The aim of the present workplace health promotion study was to evaluate the effectiveness of a targeted worksite mindfulness-related multi-component health promotion intervention on work engagement, mental health (general mental health and need for recovery) and mindfulness.

## Methods

### Design

The effectiveness of the Mindful VIP intervention was evaluated using a Randomized Controlled Trial. Participants who gave written informed consent were measured at baseline (T0), as well as 6 months (T1), and 12 months (T2) of follow-up. The study design and procedures have been approved by the Medical Ethics Committee of the VU University Medical Center. This committee gave exemption of the insurance obligation, because they judged the risk for adverse events of this intervention to be minimal. More details of the randomized controlled trial design have been described extensively elsewhere [15]. The protocol for this trial and supporting CONSORT checklist are available as supporting information; see Checklist S1 and Protocol S1. This trial was registered in the Netherlands Trial Register (NTR) (NTR2199).

### Participants

All employees from two Dutch research institutes were invited by e-mail to participate, between April 2010 and November 2010. An employee was considered eligible when having signed informed consent, not being on sick leave for more than 4 weeks, and not being pregnant at the time of recruitment. The threshold of 4 weeks sick leave is considered an indicator for long term sick leave, which could impede participation in the intervention period of six months. After enrolment, every participant remained in the study (according to the intention-to-treat principle). All participants signed informed consent. Measurement took place by appointment, in a separate room at the workplace, where the research assistant handed the questionnaire to the participant. Questionnaires and research data were only accessible for the independent research team. Employers had no access to any data. Data were coded; personal information was only connected to the data with a secured key code. Only two members of the research team had access to the key code. Small incentives (such as a ‘fit band’ or a pen) were offered for participating in each measurement. When all three measurements were completed, participants received a ‘personal report’ on request, containing their scores, and in addition they could choose between a gift certificate or one out of two mindfulness-related incentives: a meditation workshop or a chi-kung workshop.

### Randomization and blinding

After baseline measurements, participants were individually randomized to either the intervention or control group, using a computer-generated randomization sequence. After randomization, the research assistant notified each participant by e-mail about the group he or she was allocated to.

### Intervention

**Figure 1:**
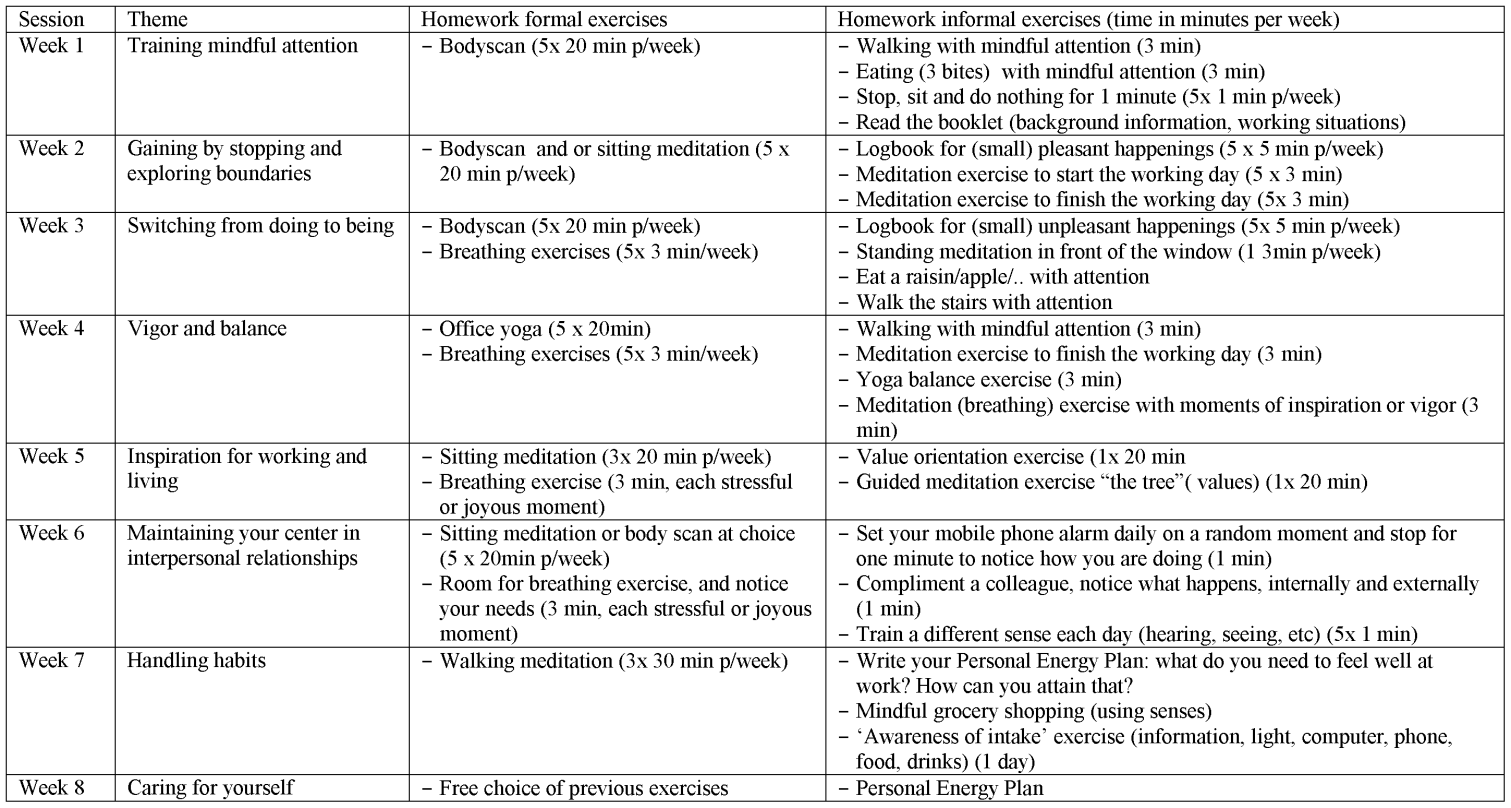
Overview of the mindfulness-based training program.

The total duration of the intervention was six months. The Mindful VIP intervention comprised 8 weeks of in-company mindfulness-related training with homework exercises, followed by 8 sessions of e–coaching. The weekly mindfulness-related training sessions took 90 minutes and were held in a room at the worksite in a group setting of 4 to 17 participants. They participated in their own time (not during paid working hours), but the timetable was adapted to working hours as much as possible (before working hours, around lunch time and after working hours). The homework exercises comprised a variety of formal (“body scan” meditation, sitting meditation) and informal exercises (small exercises, such as breathing exercises when starting up the computer, and grocery shopping mindfully) and took approximately 30 minutes per day on 5 days per week. Materials for this training consisted of 2 cd's with guided meditation exercises and a booklet with examples of workplace situations, background and (workplace) exercises. In addition, a few cognitive exercises which were hypothesized to have an effect on work engagement [6], [7] were incorporated in the training. These exercises were adjusted to the mindfulness context, such as a logbook for pleasant happenings “This week, use the log book of pleasant happenings, and notice what these happenings did, both internally and externally” (e.g. logbook for counting blessings [15]. A short overview of the mindfulness-related intervention program is presented in figure 1. The mindfulness-related training was led by four certified trainers. These trainers were all members of the Society of Mindfulness-Based trainers in the Netherlands and Flanders, which means they have followed a mindfulness trainer education that is recognized by this Society.

The e-coaching was integrated into the mindfulness-related training and was adapted to the mindfulness context as much as possible. Kindness and awareness were key-elements. During the penultimate session, the participants were asked to write a Personal Energy Plan (PEP), setting goals for themselves, answering the central question: “What do I need to do, to feel well at work?”, using the techniques and exercises from the training. (For example: ‘to sit and meditate five times a week’, or ‘to concentrate on my breath before speaking up in a meeting’.) They had to e-mail the PEP to the trainer before the last session and that marked the start of the coaching by e-mail. The trainers provided 8 e-coaching sessions, existing of positive feedback (kindness) on the PEP and answers to questions [23].

Additionally, free fruit and snack vegetables were provided during 6 months. Furthermore, lunch walking routes, and a buddy-system were offered as supportive tools. Fruit was provided at the location where the training was held. Lunch walking routes were provided by an intranet webpage. The buddy system was incorporated in the mindfulness training: the training was given in group setting and, in addition, participants were asked to form pairs to discuss homework exercises and to keep in contact between the sessions [23]. More details of the intervention and its development are described elsewhere [15].

The control intervention was offered to all participants in both the intervention and the control group. The control intervention consisted of an e-mail with a link to an internet web page. Participants in the control group did not receive anything else but the link to the web page. This web page contained information about what the organisations offered their employees with respect to health promotion. This information was already available for all employees, but for the purpose of this study, all the information about the health- and vitality related offer of the organisations was sorted together on one page. Examples of what the organisations offered with respect to health promotion were: contact information of the occupational physician and psychologist, an overview of available training and education (please note that mindfulness-related training was not provided), and information about the in-company fitness facilities. As this information was already available on the intranet, and sorting them together on one page was the only ‘intervention’, and we offered the control intervention to all participants in both the intervention and the control group, any effects of the control condition were expected to be minimal.

### Measurements

#### Work engagement

The Utrecht Work Engagement Scale (UWES) was used to measure the primary outcome of this study: work engagement. The UWES is a self-report questionnaire that measures three aspects of engagement: vigor (6 items), dedication (5 items), and absorption (6 items) [9]. Vigor refers to high levels of energy and resilience, the willingness to invest effort, not being easily fatigued, and persistence in the face of difficulties. Dedication refers to deriving a sense of significance from one's work, feeling enthusiastic and proud about one's job, and feeling inspired and challenged by it. Absorption refers to being totally and happily immersed in one's work. Answers were given on a 7-point scale from zero to six, with higher scores representing a higher level of work engagement [9]. The UWES has shown sufficient internal consistency [9]. In our study, internal consistency was excellent (Cronbach's alpha was 0.93).

#### General mental health

General mental health was measured using the corresponding items within the mental health scale from the RAND-36 [19]. Participants were asked to indicate on a six-point Likert-scale (ranging from constantly to never) for five items how often they felt anxious, depressed, calm, sad, and happy during the past four weeks. Further, the items were summed into a rough scale score, and this rough scale score was transformed into a final score on a 100 point scale, with higher scores representing better mental health [19]. The Dutch version of the RAND-36 mental health scale has shown to be sufficiently reliable [19]. In our study, internal consistency was good (Cronbach's alpha was 0.82).

#### Need for recovery

The 11-item need for recovery scale from the Dutch version of the Questionnaire on the Experience and Evaluation of Work was used [20]. These 11 items consist of statements about the recovery period after a day of work. Participants were asked to indicate whether the statement was applicable for them or not (yes/no). Examples of statements are: “My job causes me to feel rather exhausted at the end of a working day” and “After the evening meal, I generally feel in good shape”. The need for recovery scale ranges from 0 to 100 (higher scores being more unfavourable) [20]. The psychometric qualities of the need for recovery scale have shown to be sufficient [20]. In this study, internal consistency was good (Cronbach's alpha was 0.82).

#### Mindfulness

The level of mindfulness was measured using the Mindful Attention Awareness Scale (MAAS) [16]. This 15-item scale measures the frequency of everyday mindfulness experiences on a six-point scale. The psychometric qualities of the MAAS have shown to be sufficient [16]. In this study, internal consistency was good (Cronbach's alpha was 0.88).

#### Covariates

At baseline, data on potential effect modifiers were assessed, including age, gender, education (highest completed education: ‘higher vocational education/university’ or ‘other’) and marital status (‘married/significant other’ or ‘single/divorced/widow/widower’).

### Sample size

The sample size was based on finding an effect on the primary outcome of this study, work engagement, measured using the UWES [9]. An effect of a 10% increase in mean score was expected to be relevant and feasible. With a power of 90% and a two-sided alpha of 5%, both groups needed 89 participants. Accounting for a loss to follow-up of 25% over 12 months, each group needed 119 workers at baseline, thus an initial total of 238 participants for the two groups. The sample size calculation has been described more extensively elsewhere [15].

### Statistical analyses

We performed linear mixed effect models with each outcome measures as the dependent variable, group (intervention vs. control group) as independent variable and time of follow-up measurements (T1: follow up at 6 months and T2: follow up at 12 months) as fixed factor, while adjusting for the baseline levels of the outcome measure. Data were analyzed according to the intention-to-treat principle; all participants were analyzed according to the condition (i.e. intervention or control) they were initially randomized using the linear mixed effect models, despite whether, and to what extent, they were compliant to the intervention. In addition, linear regression analyses with complete case on either T1 or T2 were conducted as sensitivity analysis. All statistical analyses were performed using SPSS (Version 20, Chicago, USA). The researchers were not blinded for the analyses.

Additional post-hoc analyses were performed on subgroups based on compliance to the mindfulness-related training, as this was the main component of the intervention, and on categories of baseline work engagement scores. First, analyses were performed to gain insight into the relationship between compliance of participants to the mindfulness-related training and the effects on work engagement. Compliance categories were defined as 1) no intervention (control group), 2) low compliance: less than 6 training sessions (<75%), 3) high compliance: 6 to 8 training sessions (≥75%). To test for differences between these compliance groups, linear regression coefficients were calculated for high and low compliance, using the control group as the reference category. Second, analyses were performed on subgroups based on baseline scores of work engagement (score categories low: <4.17(median), and high: ≥4.17, under the assumption that participants with low scores of work engagement at baseline have more to gain [21]. Differences between intervention and control group were tested for both baseline score categories in (stratified) linear regression analyses.

## Results

**Figure 2:**
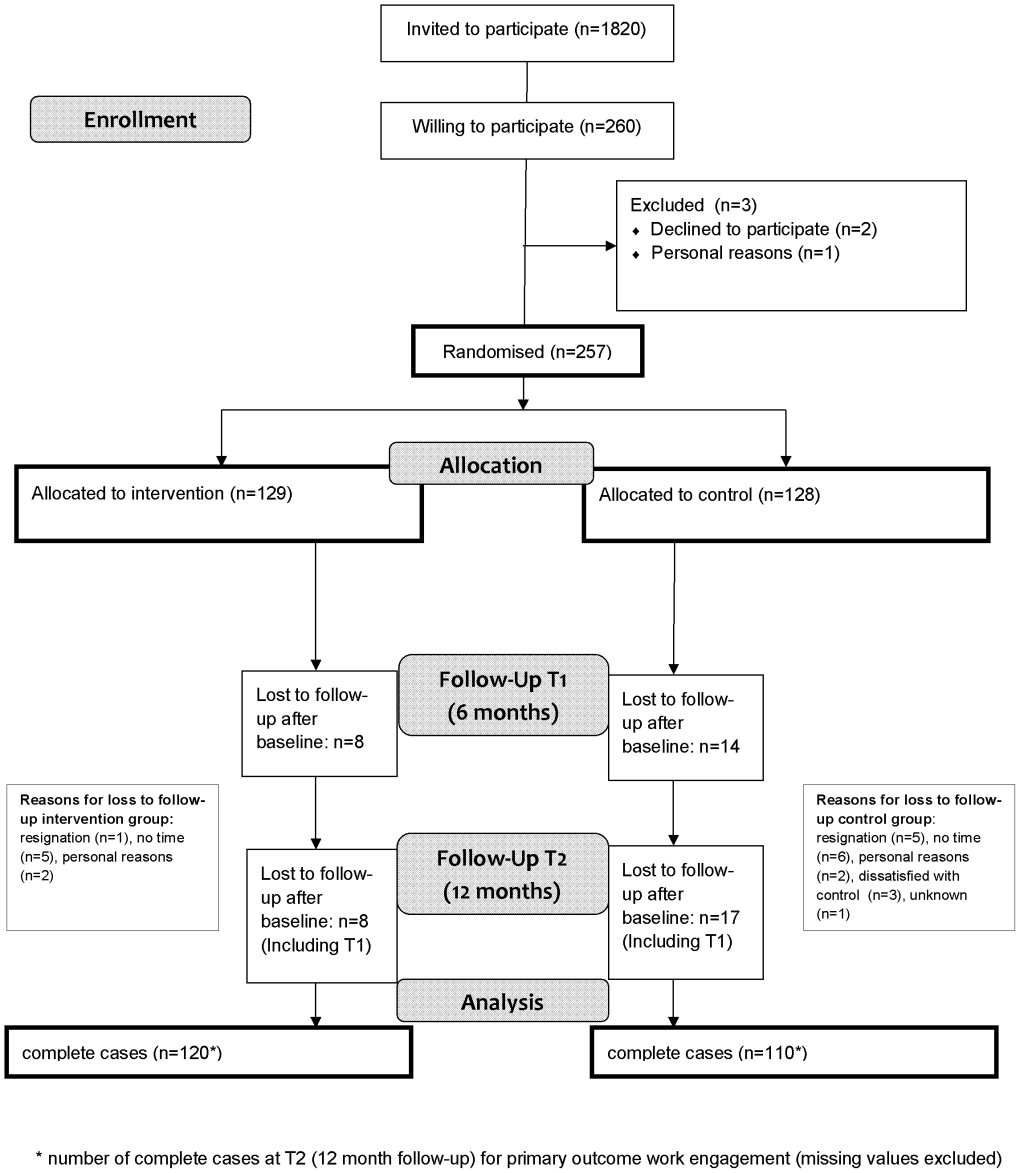
Flow Diagram Mindful VIP study.

As presented in the flow diagram of the Mindful VIP study (figure 2), a total of 257 participants completed the baseline questionnaire and were randomized to the intervention (n = 129) or control group (n = 128). Between October 2010 and November 2011, the follow-up measurements took place. After 6 months, 231 participants completed the questionnaire and after 12 months 233 participants completed the questionnaire. Loss to follow-up after 12 months was 9.1%.

In table 1, the baseline characteristics of the study population are presented. No significant differences existed between the intervention and control group. Both the intervention and the control group consisted mainly of highly educated workers (76.7% and 85.9% respectively) and of women (63.6% and 71.1% respectively). Table 2 shows the scores for work engagement, mental health, need for recovery and mindfulness at baseline and follow-up measurements after 6 and 12 months. Means for primary outcome work engagement ranged from 3.9 to 4.1 for the intervention group, and were 4.0 at all measurements for the control group. Work engagement was measured on a scale ranging from 0 to 6, with higher scores representing a more favourable work engagement. Means for secondary outcome measure mental health ranged from 73.3 to 74.8 on a scale from 0 to 100, with higher scores representing a more favourable mental health. Means for secondary outcome measure need for recovery ranged from 24.7 to 28.2 on a scale from 0 to 100, with lower scores representing a more favourable need for recovery. Means for mindfulness were for both groups at all measurements 4.0, except for the intervention group after 12 months (mean = 3.9), on a scale of 1 to 6, with higher scores representing more mindfulness. “Table 3 shows the results of the linear mixed effect models (which constitutes the primary analyses), and the results of linear regression analyses (sensitivity analyses) on work engagement, mental health, need for recovery and mindfulness. Effects in the linear mixed effect models are expressed as mean estimates (ME). This indicates the estimated effect of the intervention per follow- up measurement, corrected for baseline values. Effects in the sensitivity analyses are expressed as unstandardized regression coefficients (b). This coefficient represents the difference between the intervention group and the control group, expressed in the number of units of the scale of the dependent variable (for example 0.0–6.0 for work engagement). No significant effects of the intervention were observed after six months for work engagement (ME = −0.1 95% CI −0.1–0.2) mental health (ME = 1.3, 95% CI −0.71–3.2), need for recovery (ME = −3.0, 95% CI −6.3–0.4) and mindfulness (ME = 0.1, 95% CI −0.0–0.01). Also after twelve months, no significant effects were observed for work engagement (ME = −0.1 95% CI −0.2–0.1), mental health (ME = −1.7, 95% CI −4.6–1.1), need for recovery (ME = 2.2, 95% CI −2.5–7.0), and mindfulness (ME = −0.1, 95% CI −0.2–0.0). Results of the sensitivity analyses were similar.” Tables 4 and 5 show the results of the subgroup analyses based on the level of compliance and baseline work engagement scores. It appeared that compliance was not related to the intervention outcome. With respect to the impact of baseline work engagement, those with a low score approached significance (p = 0.053; b = −0.3) to an effect on work engagement at 12 months. No further relations were found for baseline work engagement scores and the effect on work engagement.

**Table 1:** Baseline characteristics of the Mindful VIP study (n = 257).

	Intervention group(n = 129)	Control group(n = 128)
Demographics		
Gender: Female, %	63.6	71.1
Marital status: Married or significant other, %	81.4	73.4
Education: Highly educated*, %	76.7	85.9
Age in year*s*, mean (sd)	46.0 (9.4)	45.1 (9.6)

* Higher vocational education or university.

**Table 2:** Scores on work engagement, mental health, need for recovery and mindfulness at baseline (T0), follow-up at six (T1) and 12 months (T2) for intervention and control group.

	Group	T0 Mean(sd)	n	T1 Mean(sd)	n	T2 Mean (sd)	n
Work engagement	I	4.1 (0.8)	129	4.0 (0.9)	115	3.9 (0.9)	120
(Range: 0–6)	C	4.0 (0.9)	126	4.0 (0.9)	108	4.0 (0.9)	112
Mental Health	I	74.8 (12.9)	129	74.8 (12.2)	116	73.3 (13.8)	119
(Range: 0–100)	C	73.6 (14.1)	127	74.5 (14.1)	109	74.6 (13.9)	111
Need for recovery	I	26.0 (24.0)	126	24.7 (23.9)	116	27.4 (26.1)	117
(Range: 0–100)	C	28.2 (27.8)	126	25.7 (24.2)	111	26.5 (27.1)	109
Mindfulness	I	4.0 (0.6)	126	4.0 (0.6)	115	3.9 (0.6)	117
(Range: 1–6)	C	4.0 (0.7)	127	4.0 (0.8)	109	4.0 (0.7)	111

I = Intervention C = Control.

**Table 3:** Intervention effects on work engagement, mental health, need for recovery and mindfulness after 6 (T1) and 12 months (T2), corrected for baseline values (T0); results from linear mixed effect models (primary analyses) and linear regression models (sensitivity analyses).

Primary analyses			T1	T2
	Group	n	ME	p-value	95%CI	n	ME	p-value	95% CI
**Work engagement**	**I**	**115**	−0.1	0.33	−0.1–0.2	**120**	−0.1	0.48	−0.2–0.1
**(Range: 0–6)**	**C**	**107**				**110**			
**Mental Health**	**I**	**116**	−1.2	0.21	−0.7–3.2	**119**	−1.7	0.23	−4.6–1.1
**(Range: 0–100)**	**C**	**109**				**111**			
**Need for recovery**	**I**	**113**	−3.0	0.08	−6.3–0.4	**115**	2.2	0.36	−2.5–7.0
**(Range: 0–100)**	**C**	**110**				**108**			
**Mindfulness**	**I**	**113**	0.1	0.12	0.0–0.1	**115**	−0.1	0.23	−0.2–0.0
**(Range: 1–6)**	**C**	**109**				**111**			

Sensitivity analyses			T1	T2
	Group	n	b	p-value	95%CI	n	b	p-value	95% CI
**Work engagement**	**I**	**115**	−0.1	0.43	−0.3–0.1	**120**	−0.1	0.10	−0.3–0.0
**(Range: 0–6)**	**C**	**107**				**110**			
**Mental Health**	**I**	**116**	−0.5	0.68	−3.1–2.2	**119**	−2.6	0.06	−5.2–0.1
**(Range: 0–100)**	**C**	**109**				**111**			
**Need for recovery**	**I**	**113**	−1.5	0.52	−6.1–3.1	**115**	1.3	0.61	−3.7–6.3
**(Range: 0–100)**	**C**	**110**				**108**			
**Mindfulness**	**I**	**113**	0.0	0.61	−0.1–0.1	**115**	−0.1	0.37	−0.2–0.1
**(Range: 1–6)**	**C**	**109**				**111**			

I = Intervention C = Control, CI = Confidence Interval, ME = Mean Estimate of adjusted effects b = unstandardized regression coefficient.

**Table 4:** Effects of the intervention on work engagement in subgroups: low and high compliance to the mindful VIP intervention compared to the control group as reference category.

		T1	T2
Group		b	p-value	95%CI	b	p-value	95% CI
Control		Reference			Reference		
Intervention	Low compliance	−0.1	0.57	−0.6–0.3	0.0	0.93	−0.4–0.4
Intervention	High Compliance	0.0	0.87	−0.5–0.4	−0.1	0.77	−0.5–0.3

CI = Confidence Interval, b = unstandardized regression coefficient.

**Table 5:** Effects of the intervention on work engagement in subgroups: stratified analyses for low and high work engagement at baseline.

Group		T1	T2
		b	p-value	95%CI	b	p-value	95% CI
Baseline Work engagement score	Low (n = 127)	−0.1	0.41	−0.4–0.2	−0.3	0.05*	−0.5–0.0
	High (n = 128)	0.0	0.99	−0.3–0.3	0.0	0.94	−0.2–0.2

* P = 0.053, CI = Confidence Interval, low≤4.17 (cut-off) high>4.17(cut-off), b = unstandardized regression coefficient.

## Discussion

No intervention effects were observed for work engagement, mental health, need for recovery and mindfulness after 6 and 12 months. Furthermore, this study showed no relationship between compliance to the mindfulness-related training and intervention effects. Intervention effects were not different between subgroups of high or low engagement at baseline.

We expected, based on literature that an increase in mindfulness would lead to an increase in work engagement [6], [7]. Our intervention did not lead to an increase in mindfulness, which hampers the possibility to gain insight into the effects of an increase in mindfulness on work engagement scores. This also applies to the effects of an increase in mindfulness on mental health and need for recovery.

A possible explanation for the lack of effect of our worksite mindfulness-related multi-component intervention is the intensity and duration of our training component of our intervention. Traditionally, mindfulness training consists of 8 to 10 weekly sessions of 2.5 to 3 hours [18]. In the present study, in consultation with the target population, maximum 8 weekly sessions with a duration of maximum 1.5 hours were considered feasible. It might be that the mindfulness-related training we designed for this study was not sufficiently intensive to generate effects, measured at six and twelve months after baseline. Moreover, our training did not comprise only mindfulness exercises, but consisted for a small part of other cognitive activities, adapted to the mindfulness context, such as complimenting colleagues and noticing what happens internally and externally. This possibly made the mindfulness-related training component even less intense. However, another study [18] with an even less intensive worksite program (6 weekly sessions of one hour) did report beneficial - though immediate- - effects on mindfulness, also measured using the MAAS.

Next to the limited intensity and duration of the training component, the lack of finding the expected effects might also explained by characteristics of the study population and design. Reviews show that the majority of mindfulness-related interventions are evaluated among patient groups (for example groups suffering from chronic pain), aiming at reducing symptoms rather than primary prevention or health promotion [17]. Moreover, previous randomized controlled studies on mindfulness were carried out in experimental settings involving smaller populations (range n = 22–n = 97) [17], [18], [22], and randomized controlled designs are not common [17]. In addition to the study design and population, also the timing of the measurements could explain why we did not find the expected effects after six and twelve months. Previous studies examined effectiveness of mindfulness-related training prevailingly immediately after the training. A meta-analysis [17] of both controlled and observational studies examining mindfulness, only included immediate pre- to post intervention measurements, i.e. 6 to 8 weeks, of (absence of) mental disorders. Respondents of interviews from the process evaluation of the mindful VIP intervention [23] reported feeling ‘revitalized’, ‘fresh’, ‘energetic’, and ‘peaceful’ immediately after a training session, but they also reported that this faded away [23]. It might be that the mindfulness-related training only had an immediate effect, which could only have been captured directly after the training and not after 6 or 12 months. It was intended to prolong mindfulness practice through e-coaching by the mindfulness trainers. However, as only 6.3% of the participants complied with the dose of e-coaching as intended [23], we are impeded to make statements on the effects of prolonged mindfulness practice. The low compliance with e-coaching might explain the lack of effect on mindfulness (and other outcomes) after 6 and 12 months.

Another possible explanation for not finding an effect might be the occurrence of cross-over of effects. These effects may have decreased the contrast between intervention and control groups. However, in our study, cross-over effect do not seem likely as the control group had no access to the training and e-coaching. It could however be, that the other elements (fruit, lunch walking, buddy system) were used by the control condition. Yet, the means scores on the outcome measures stay stable for both groups and when cross-over effects would have taken place, a slight increase in mean scores of both groups would be expected. Therefore, although we did not measure cross-over effects (nor use of the control condition) in the control group, the likelihood of cross-over effects seem minimal.

Although in the present study increasing mindfulness was the main strategy of increasing work engagement, also other strategies were incorporated in the intervention, such as goal setting and positive reinforcement in the e-coaching [15]. Therefore, also without an increase in mindfulness, hypothetically, there could have been an effect on work engagement. However, since there was a lack of effect on work engagement, this was not the case.

Since mindfulness is not a goal-oriented state but an open and accepting state, our choice for goal setting and related strategies might raise questions on the expected direction of effects. However, since we chose to set goals in the context of mindfulness practice, we adapted these strategies to match with mindfulness principles (see figure 1). For example, a participant wanted to continue mindfulness practice when leaving for a holiday, but found it difficult to find time and to sit and meditate with all the packing and preparing chores. The e-coach gave ideas on how to incorporate mindfulness practice while packing (stop a few times to feel your breath when packing your bags) and other preparing tasks. In the following e-mail session, the participant reflected on how that went.

Two previous studies have also examined the effectiveness of a workplace intervention on work engagement: a web-based self-enhancement intervention, consisting of happiness assignments, goal setting assignments and resource building assignments (21), and a worksite intervention consisting of yoga, workout and coaching (24). However, these studies did not find an overall effect on work engagement as well [21], [24]. Unlike this study, the self enhancement intervention [21] found an increase of work engagement within the subgroup that scored low on work engagement at baseline. Also, the authors reported an effect on assumed precursors (positive emotions and self-efficacy) of work engagement, which were not measured in the present study. The two previous studies [21], [24], together with the present study, were aimed at the individual aspects. Work engagement is, however, not only hypothesized to be determined by individual factors, but also by environmental factors [6], [7], [25]. These individual factors are the ‘personal resources’ in the Job Demands and Resources (JDR) model [26]. Personal resources are considered to be easiest to influence in an intervention. In addition, personal resources are considered important as they cause different personal reactions to the same organisational environment [6], [7]. The organisational environmental factors are ‘job demands’ and ‘job resources’ in the JDR model [26]. Job resources and personal resources are mutually related [26]. As this study aimed only minimally at job resources (i.e. social support), it might be that not taking into account the job resources contributes to the lack of effect.

Another explanation for the lack of effects, might be that a so-called ‘ceiling effect’ occurred. This would be the case when the baseline scores were high to such an extent, that improvement is practically not possible. For baseline scores of work engagement however, it appeared that the mean score of our study population (4.1 on a scale of 0 to 6) falls in the category ‘average engaged’(range 3.1–4.7), compared to the Dutch norm scores with the same 17-item scale [9]. Although the mean score was somewhat higher in our study population (4.1) than in the Dutch population (3.8) [9], we consider the possibility of a ceiling effect not to be the primary cause of the lack of effects because their seems to be room for improvement. Also, the scores of our study population for mental health and need for recovery (74 and 27 respectively) are well comparable to Dutch norm scores (77 and 27 respectively) [19], [20]. In addition, the mean score for mindfulness on the Mindful Attention and Awareness Scale (4.0) is well comparable to a control group (4.0) in the validation paper of that scale [16].

In the subgroup with low work engagement scores at baseline, the intervention pointed in the direction of a (non significant) negative effect on work engagement. In the entire study population, the intervention pointed to a (non-significant) negative effect on mental health after twelve months. We cannot rule out that these findings did not reach significance due to lack of power, as the power calculation was based on finding an effect on work engagement in the entire study population. However, as the effect in both cases was smaller than 10%, the findings are considered as not relevant.

The major strength of this study is the randomized controlled trial design, which is the most reliable design for intervention studies. Second, the duration of follow-up was 12 months, which can be considered quite long and unique for mindfulness-related research, in which follow-up measurements usually take place directly after completing the training only [17], [18], [22]. Another strength, is that the intervention was tailored to the needs of the target population. Furthermore, this study is the first to study the effectiveness of mindfulness-related training on work engagement. In addition, loss to follow-up was very limited (less than 10%), as we had taken into account a 25% loss to follow up in our sample size calculation [15].

A limitation of this study was that environmental factors have not been taken into account. This intervention targeted individual aspects of work engagement only because these were hypothesized to be most susceptible to change and to be discriminating factors in why different workers react differently to the same environmental factors, at the time of the development of this intervention [7]. However, not only is the effectiveness of targeting a combination of individual and job aspect probably more effective (as argued above), it is also questionable whether it is ethically sound to target interventions that are potentially beneficial for organisations solely at the individual. Second, no precursors of work engagement were measured, because of which no early changes in work engagement could be detected. The two last limitations have to do with the population under study and the fact that the intervention is specifically developed to meet their needs, limiting the generalizability of the results. Women and highly educated workers [23] were over represented compared to the source population (employees of the participating two research institutes). In addition, as the content of the intervention was adjusted to the target population of workers with scientific professions, the findings of this study are only limited to this professional group.

For future multi-component intervention research, it is recommended to perform follow-up measurements directly after each component, as well as on the longer term, to gain insight in the preservation of possible effects. For future work engagement research, it is recommended to intervene on environmental determinants (job resources) of work engagement, or at the combination of individual and job environment. As job and personal resources are mutually related [8] and studies examining the effect of personal resources alone found no effects on work engagement, job resources or a combination of job and personal resources seem to be most promising. It is also recommended to use larger study populations to allow subgroup analyses with sufficient power. For future implementation of mindfulness-related interventions, it is recommended to increase the compliance to e-coaching or other ways to continue the mindfulness principles, to maintain immediate beneficial effects, if present.

## Conclusion

Although evidence on the immediate effects of mindfulness-related training exists for various mental well-being and health outcomes, the results of this study showed no effects of a targeted mindfulness-based intervention, consisting of a mindfulness-related training, e-coaching, lunch walking, free fruit and a buddy system, on work engagement, mental health, need for recovery and mindfulness after 6 and 12 months. The lack of finding an increase in mindfulness in this study impedes us from making statements about the possible relationship between increasing mindfulness and increasing work engagement.

## Supporting Information

Checklist S1 **CONSORT Checklist.**
(DOC)

Protocol S1 **Trial Protocol.**
(PDF)

Protocol S2 **Study protocol IRB.**
(PDF)
