# Correction: Effectiveness of a Worksite Mindfulness-Related Multi-Component Health Promotion Intervention on Work Engagement and Mental Health: Results of a Randomized Controlled Trial

**DOI:** 10.1371/journal.pone.0122428

**Published:** 2015-03-26

**Authors:** The PLOS ONE Staff

There is an error in the fifth to the last sentence of the Results. The correct sentence is: No significant effects of the intervention were observed after six months for work engagement (ME = 0.1 95% CI 0.1–0.2) mental health (ME = 1.3, 95% CI 0.7–3.2), need for recovery (ME = −3.0, 95% CI −6.3–0.4) and mindfulness (ME = 0.1, 95% CI −0.0–0.01).

There are errors in Table 3. Please see the corrected Table 3 here.

**Table 3:** Intervention effects on work engagement, mental health, need for recovery and mindfulness after 6 (T1) and 12 months (T2), corrected for baseline values (T0); results from linear mixed effect models (primary analyses) and linear regression models (sensitivity analyses).

Primary analyses			T1				T2		
	Group	n	ME	p-value	95%CI	n	ME	p-value	95% CI
**Work engagement**	**I**	**115**	0.1	0.33	-0.1–0.2	**120**	-0.1	0.48	-0.2–0.1
**(Range: 0–6)**	**C**	**107**				**110**			
**Mental Health**	**I**	**116**	1.2	0.21	-0.7–3.2	**119**	-1.7	0.23	-4.6–1.1
**(Range: 0–100)**	**C**	**109**				**111**			
**Need for recovery**	**I**	**113**	-3.0	0.08	-6.3–0.4	**115**	2.2	0.36	-2.5–7.0
**(Range: 0–100)**	**C**	**110**				**108**			
**Mindfulness**	**I**	**113**	0.1	0.12	0.0–0.1	**115**	-0.1	0.23	-0.2–0.0
**(Range: 1–6)**	**C**	**109**				**111**			

Sensitivity analyses			T1				T2		
	Group	n	b	p-value	95%CI	n	b	p-value	95% CI
**Work engagement**	**I**	**115**	-0.1	0.43	-0.3–0.1	**120**	-0.1	0.10	-0.3–0.0
**(Range: 0–6)**	**C**	**107**				**110**			
