# Supplementary material for: Effectiveness of a Worksite Mindfulness-Related Multi-Component Health Promotion Intervention on Work Engagement and Mental Health: Results of a Randomized Controlled Trial
Source: PLoS One. 2014 Jan 28;9(1):e84118. doi: 10.1371/journal.pone.0084118 (PMC3904825; doi:10.1371/journal.pone.0084118)
Supplement: Protocol S2 — Study protocol IRB. (PDF) [file pone.0084118.s003.pdf]

VIP in Onderzoek

The (cost-)effectiveness of an intervention on energy balance related behaviours and work engagement

Department of Public and Occupational Health
EMGO Institute for Health and Care Research
VU University medical Center
Van der Boechorststraat 7
1081 BT Amsterdam

- ☏: +31 20 444 58 55
- ✉: j.vanberkel@vumc.nl
- www.vitaalinpraktijk.nl

Jantien van Berkel MA
Dr. Cécile Boot
Dr. Karin Proper
Prof. dr. ir. Paulien Bongers
Prof. dr. Allard van der Beek

Protocol ID	
Short title	VIP in Onderzoek
Version	1
Date	
Promotores	Prof. AJ. van der Beek, PhD<br>Department of Public and Occupational Health<br>EMGO Institute for Research in Health and Care<br>VU University Medical Center<br>Van der Boechorststraat 7<br>1081 BT Amsterdam, the Netherlands<br><br>Prof. P. M. Bongers, PhD<br>TNO Quality of life<br>Hoofddorp
Co-promotor, project leader and daily supervisor	KI. Proper, PhD<br>Department of Public and Occupational Health<br>EMGO Institute for Research in Health and Care<br>VU University Medical Center<br>Van der Boechorststraat 7<br>1081 BT Amsterdam, the Netherlands<br><br>CRL. Boot, PhD<br>Department of Public and Occupational Health<br>EMGO Institute for Research in Health and Care<br>VU University Medical Center<br>Van der Boechorststraat 7<br>1081 BT Amsterdam, the Netherlands
Principal investigator	J. van Berkel, MA<br>Department of Public and Occupational Health<br>EMGO Institute for Research in Health and Care<br>VU University Medical Center<br>Van der Boechorststraat 7<br>1081 BT Amsterdam, the Netherlands
Sponsor (in Dutch: verrichter/opdrachtgever)	Delta Lloyd/Ohra, Zorgverzekeraar
Independent physician	Pieter Gallee, MD<br>Arbeid & Milieu Dienst<br>VU University Medical Center<br>Van der Boechorststraat 1<br>1081 HV Amsterdam, the Netherlands

PROTOCOL SIGNATURE SHEET

**Name**	**Signature**	**Date**
Prof. dr. Allard van der Beek<br>Promotor<br><br>Prof. dr. ir. Paulien Bongers<br>Promotor		
Dr. Karin Proper<br>Copromotor and project leader<br><br>Dr. Cécile Boot<br>Copromotor		
Jantien van Berkel MA<br>Principal investigator		

Table of contents

PROTOCOL SIGNATURE SHEET......3
1. Introduction and Rationale......6
1.2 Objectives......7
1.3 Research questions......7
2. Study design and phasing......8
2.1 Phase 1: development of the lifestyle intervention......8
2.1.1 Step 1: Needs assessment......8
2.1.2 Step 2: Definition of performance objectives......12
2.1.3 Step 3: Methods & Strategies......12
2.1.4 Step 4: Program Development (November – December 2009)......16
2.1.5 Step 5: Adoption and implementation (January 2010 – January 2011)......16
2.1.6 Step 6: Evaluation......16
2.2 Phase 2: Evaluation......16
2.3 Intervention......17
2.3.1 Mindfulness......17
2.3.2 E-coaching......18
2.3.3 Supporting elements......19
3. Study Population......19
3.1 Population......19
3.2 In- and exclusion criteria......19
3.3 Sample size calculation......19
4. Methods......20
4.1 Recruitment......20
4.2 Randomisation......21
4.3. Measurements......21
4.3.1 Primary endpoints......21
4.3.1.1 Energy balance related behaviours......21
4.3.1.2 Work engagement......22
4.3.2 Secondary endpoints......22
4.3.3 Other endpoints......23
4.3.4 Data analysis......24
4.3.4.1 Statistical analysis......24
4.3.4.2 Economic evaluation......24
5. Study procedures......26
5.1 Withdrawal of individual subjects......26
5.1.1 Specific criteria for withdrawal......26
5.1.2 Replacement of individual subjects after withdrawal......26
5.1.3 Follow-up of subjects withdrawn from treatment......26
5.1.4 Premature termination of the study......26
6 Safety reporting......27
6.1 Section 10 WMO event......27
6.2 Adverse and serious adverse events......27

6.3 Follow-up of adverse events 27
6.4 Data Safety Monitoring Board (DSMB) 27
7. Ethical considerations 27
7.1 Regulation statement 28
7.2 Recruitment and consent 28
7.3 Benefits and burden assessment 28
7.4 Compensation for injury 28
7.5 Compliance and incentives 29
8. Administrative aspects and publication 29
8.1 Handling and storage of data and documents 29
8.1.2 Data assessment 29
8.1.3 Data entrance 30
8.1.4 Data storage 30
8.2 Amendments 30
8.3 Annual progress report 30
8.4 End of study report 30
References 31
Appendices 36

1. Introduction and Rationale

With strongly developing technology and welfare, daily life in the western European countries has changed tremendously over the last decades. Work has also consequently changed: a shift is noticeable from more physically active work to more sedentary work because of automation. The change in daily life and work makes it unnecessary to be physically active. This inactiveness results in a positive energy balance – together with the availability and growing portion size of foods, amongst other reasons - and it is an important cause of weight gain (Hill, 2006). Weight gain in its turn leads to overweight.

As overweight has grown in prevalence over the last few decades (Visscher & Seidell, 2004; WHO,2004), it is considered a growing threat to the health of the working population. It is linked – together with obesity- to an increased risk of numerous severe diseases (WHO, 2004) and thereby increased health care costs (Seidell, 1998). In addition, it has been shown that obese workers have higher sick leave and especially sick leave of a longer duration (Van Duijvenbode et al, 2009; Neovius et al, 2009). Overweight is also associated with lower levels of productivity (Pronk et al., 2004; Hertz et al., 2004). In short, preventing overweight is beneficiary from the worker perspective but also from the company perspective.

To prevent overweight, both sides of the energy balance should be targeted: energy expenditure (i.e. physical activity behaviour) and energy intake (i.e. dietary behaviour). Besides physical activity, sedentary behaviour is increasingly independently associated with overweight and obesity (Proper et al, 2007). The effectiveness of work interventions on increasing physical activity have been reported in multiple meta-analytic reviews (Dugdill et al, 2008; Bravata et al, 2007). Worksite interventions on environmental changes can effectively influence dietary intake (Engers et al, 2005). Most research about the relationship between energy balance related behaviours and health focuses on people who are already overweight or obese (Ross et al, 2009; Kaukua et al, 2003; Kaukua et al, 2002; Rippe et al, 1998). However, the evidence towards effective interventions aimed at the prevention of overweight is limited (Lombard et al, 2009: Lemmens et al, 2008).

Next to overweight, another concept that has become more important and relevant for the health of the working population over the last few years is work engagement. Work engagement has been defined as a “positive, fulfilling, work related state of mind that is characterised by vigour [vitality], dedication and absorption” (Schaufeli et al. 2002, 3:71-92). Employees with a high level of work engagement have shown to score low on burnout, anxiety and depression (Gonzalez-Roma, Schaufeli, Bakker & Lloret, 2006; Schaufeli, Taris

& Van Rhenen, 2008). Furthermore, work engagement has shown to negatively predict absenteeism frequency and absenteeism duration in a recent longitudinal study among telecom workers (Schaufeli, Bakker & Van Rhenen, 2009). In addition, other recent studies have shown that higher levels of work engagement were related to higher financial turnover (Xanthopoulou et al., 2009) and job performance (Demerouti & Bakker, 2006). In short, stimulating work engagement not only has possible benefits for individual well-being but also for business related performance outcomes.

1.2 Objectives

The current study aims to 1) develop an intervention to improve workers' energy balance related lifestyle behaviours (EBRB) to prevent overweight and to improve work engagement and 2) subsequently evaluate the intervention developed.

1.3 Research questions

Questions that need to be answered to reach the objective of this study are:

1. What is the content and format of a potentially effective intervention on EBRB and work engagement among employees of a Dutch research organisation?
2. What is the effect of the tailored intervention on the EBRB and work engagement of the employees?

2. Study design and phasing

This study consists of the development and evaluation of an intervention aimed at improving EBRB and work engagement in a worker population that is characterised by a large proportion of highly educated office workers. The Intervention Mapping (IM) approach (Bartholomew et al., 2006) has been used to ensure a systematic construction of the EBRB intervention in which scientific evidence and practice based information is combined. The intervention will be evaluated in a randomised control trial (RCT). Participants will be measured at baseline (T0), after 6 months (T1) and after 12 months (T2).

2.1 Phase 1: development of the lifestyle intervention

The intervention has been developed by means of IM. It describes a stepwise process for theory- and evidence-based development of a health promotion intervention. Intervention mapping consists of six steps: 1) needs assessment, 2) definition of program objectives, 3) selection of adequate theories and methods to realise behavioural change, 4) program development, 5) development of a plan for implementation and 6) evaluation.

2.1.1 Step 1: Needs assessment

In the needs assessment, the EBRB, work engagement and their associated determinants among the target group were assessed. This was done by 1) thorough literature search, 2) interviews with key figures, 3) a brief questionnaire and 4) focus group interviews with employees.

1) Literature search

Overweight, work engagement and their prevalence were assessed by a thorough literature search in Picarta, PubMed, Psychinfo and Sport Discus. These concepts were assessed in relation to specific characteristics of the target group, such as work setting and high level of education. In addition, determinants of EBRB and work engagement were explored.

Overweight and obesity are commonly measured by the body mass index (BMI). BMI is calculated by dividing body weight in kilograms by the square of the body height in meters. Average BMI for the Dutch working population appeared to be 24,3 kg/m^2. 31% of the Dutch working population is overweight (BMI 25.0-29.9 kg/m^2) and 6% is obese (BMI ≥ 30.0 kg/m^2) (Proper & Hildebrandt, 2009, p61). Furthermore, it appeared that women had a lower BMI than men and higher educated workers have a lower BMI than low educated workers. Other

differences in prevalence of overweight and obesity existed between occupational groups and sectors. Almost all groups professions are represented within the organisation of this study but scientific professions more than others. Workers with scientific professions had the lowest average BMI and a relatively low prevalence of overweight (26%) and obesity (4%) according to Proper & Hildebrandt (2009). These professions are not of the most physical type, which indicates that not only physical activity plays a role but also other factors such as dietary intake. The sector to which the organisation of our study belongs to is governmental. According to Proper & Hildebrandt (2009), this sector had an average BMI of 24.2. 29% of workers in this sector were overweight and 5% of them was obese.

Work engagement is measured by the UWES (Schaufeli & Bakker, 2002). Mean score for work engagement is 3,82 (on scale from zero to six, where 0 is the most negative and 6 the most positive). Occupational professions how scored highest in work engaged are farmers (M=4.24), managers (M=4.22) and office workers in a profit organisation (M= 3.97). Occupational professions how scored lowest in work engagement are gendarmes (M= 3.69), production worker (M=3.63) and doctors (who had career doubts) (M= 3,10).

2) Interviews with key figures
Face-to-face interviews were held with 6 key figures within the participating organisation to gain a first insight into organisational culture and employee characteristics in terms of EBRB issues and work engagement.

3) Brief questionnaire

A brief questionnaire was held in order to determine the main EBRB problems and status of work engagement. Thus, the questionnaire included questions about diverse sub behaviours of physical activity (at work, in leisure time, during commuting, etc) and sedentary behaviour (at work and in leisure time), dietary behaviour (vegetables and fruit intake, snacking, soda and alcohol intake, fast food and ready cooked meals). Furthermore, the questionnaire contained questions about work engagement (vigour, absorption and dedication) and questions about work recovery need as this correlates with psychological distress (Jansen et al, 2002) and burnout (Sluiter, Van der Beek & Frings-Dresen, 1999).

The brief questionnaire was sent to 100 employees of the participating organisation. They were approached by their supervisors, who were selected from different departments in order to get a representative sample. Each supervisors randomly selected four employees to

complete the questionnaire. Of the 100 employees approached, 78 completed and returned the brief questionnaire.

Based on the interviews with key figures and the questionnaires the following health related problems were identified:

- Physical activity:
    - insufficient levels of leisure time physical activity
    - sedentary behaviour during working time
- Dietary behaviour:
    - not eating the daily recommended amount of fruit and vegetables
- Work engagement:
    - low work engagement

4) Focus group interviews with workers
Based on the outcomes of the preceding step, two different semi-structured focus group interview guides were composed. The first one consisted of questions about the problems in physical activity and dietary behaviour, the second one focussed on work engagement. Interview guides were used during the focus group interviews.

In total, six focus groups (three of the first type and three of the second type) were carried out among 39 workers of the participating organisation. The aims of the focus group interviews were:

1) - identifying key determinants of the energy balance related behaviours
   - identifying ideas about how lifestyle can be improved
2) - identifying key determinants of work engagement
   - identifying ideas about how work engagement can be improved

Participants were asked to think of determinants and to write them on post-its (one keyword per post-it). The post-its were then pasted on a flap over so aal participants were able to read what everybody had written down. Subsequently, the determinants were discussed, first in an exploratory manner to discover which problems were underneath. Next, solutions or opportunities from the employees' point of view were discussed.

Based on literature, interviews and focus groups, the most important determinants of **leisure physical activity of vigorous intensity** were: perceived behavioural control, perceived barriers (especially lack of time), social support and intention.

The most important determinants for **sedentary behaviour at work, including during lunchtime** were: perceived behavioural control, perceived barriers, awareness, social support and physical environment.

The most important determinants for **vegetable and fruit intake** were: habit, perceived behavioural control, availability, costs and intention.

According to the interviewees, physical activity of vigorous intensity during leisure time can be improved by:

- time management to incorporate time for exercise in daily schedule
- goal setting (training for a marathon or game or reaching a destination)
- health check to increase awareness.

According to the interviewees, sedentary behaviour at work can be improved by:

- walking during lunch break
- changing the norms at work such that walking during lunch is acceptable or even common or desirable
- raising awareness about sedentary behaviour.

The interviewees indicated that vegetable and fruit intake can be improved by:

- providing an easier access to fruit/ vegetables
- increasing knowledge about preparing food (vegetables)
- translating healthy dietary behaviour into short term benefits
- information about nutritional values in the canteen.

The last three focus groups concentrated on identifying determinants of work engagement. The format of these interviews was about the same as the first three.

Based on literature, interviews and focus groups, the most important determinants of work engagement are: perceived behavioural control, (organisational based) self-esteem, optimism, perceived barriers and social support.

According to the interviewees, work engagement can be improved by:

- goal setting
- ‘self realisation’, by learning to be more positive, assertive and self confident and to search for possibilities within limitations and to gain more insight in the own possibilities and skills

- participate in courses and training provided by 'Bureau Talent'
- create more awareness of the possibilities that already are available
- train managers in effective leadership and communication
- change social climate to be less individualistic.

Conclusion

The focus group interviews confirmed the assumption that EBRB and work engagement appeared to be allied for the target group.

Identified factors that might threaten health were insufficient levels of physical activity during leisure time, much time spent on sedentary behaviour at work, the low levels of intake of fruit and vegetables. Additionally, the target group was identified as rather low work engaged.

2.1.2 Step 2: Definition of performance objectives

Based on the results of step 1, performance objectives were defined. These provide the foundation for the intervention by specifying who and what will change as a result of the intervention. The formulated programme objectives were:

- increasing vigorous physical activity in leisure time
- reducing sedentary behaviour at work, including lunchtime
- increasing fruit and vegetable intake
- increasing work engagement

2.1.3 Step 3: Methods & Strategies

In step 3, theory-informed methods and practical strategies were chosen to improve the selected lifestyle behaviours and work engagement of the target group. A method is a theory-based technique to influence determinants of behaviour or environmental conditions, whereas a strategy is a way of organising and operationalising the intervention methods. Methods and strategies were chosen based on the key determinants selected in step 1.

Table 1 Methods and strategies selected for increasing physical activity in leisure time of vigorous intensity

Determinants	Theoretical method	Practical strategy	Tools and Materials
**Perceived behavioural control**	Goal setting	Facilitate formulation of goals and reflection	E-coaching: Employees formulate goals and reflect in personal e-log-book on website. Coach reads and stimulates reflection.
	Reinforcement	Provide positive	E-coaching: coach provides positive feedback

		feedback	
**Perceived barriers**	Goal setting	Facilitate formulation of goals and reflection	E-coaching: Employees formulate goals and reflect in e-log-book on website. Coach reads and stimulates reflection by providing feedback.
**Social support**	Mobilising social support	Form duo's or groups for exercising and commuting to work by bike	Added to the already existing carpool system: a bike pool system to commute to work and a buddy system for exercising.
**Intention**	Self regulation	Teach skills	Incompany mindfulnesstraining with exercises aimed at enacting on intentions

Table 2 Methods and strategies selected for reducing sedentary behaviour at work

**Determinants**	**Theoretical method**	**Practical strategy**	**Tools and Materials**
**Perceived behavioural control**	Goal setting<br><br>Reinforcement	Facilitate formulation of goals and reflection<br><br>Provide positive feedback	E-coaching: E formulate goals and reflect in personal e-log-book on website. Coach reads and stimulates reflection.<br><br>E-coaching: coach provides positive feedback
**Perceived barriers**	Goal setting<br><br>Reinforcement	Facilitate formulation of goals and reflection<br><br>Provide positive feedback	E-coaching: E formulate goals and reflect in personal e-diary on website. Coach reads and stimulates reflection.<br><br>E-coaching: coach provides positive feedback
**Awareness**	Self monitoring<br><br>Self evaluation<br><br>Self regulation	Facilitate and stimulate monitoring and evaluation<br><br>Teach skills	E monitor behaviour in a personal e- log-book<br><br>E-coaching: E formulate goals and reflect in personal e-log book on website. Coach reads and stimulates reflection.<br><br>Incompany mindfulnesstraining with exercises aimed at body awareness
**Social support**	Mobilising social support	Form duo's or groups for lunch walks	Buddy system for lunch walking (on the intranet)
**Physical environment**	Environmental change	Facilitation of reducing sedentary behaviour	Providing routes for lunchwalking

Table 3 Methods and strategies selected for increasing fruit and vegetable intake

Determinants	Theoretical method	Practical strategy	Tools and Materials
**Habit**	Self regulation	Teach skills	Incompany mindfulness training with exercises aimed at preventing counter intentional habits from obstructing enactment of intentions
	Goal setting	Facilitate formulation of goals and reflection	E-coaching: Employees formulate goals and reflect in personal e-diary on website. Coach reads and stimulates reflection
	Reinforcement	Providing positive feedback	E-coaching: coach provides positive feedback with regard to set goals
**Perceived behavioural control**	Self regulation	Teach skills	Incompany mindfulness training with goals, barriers and actions exercise
	Goal setting	Facilitate formulation of goals and reflection	E-coaching: Employees formulate goals and reflect in personal e-diary on website. Coach reads and stimulates reflection.
	Reinforcement	Provide positive feedback	E-coaching: coach provides positive feedback
**Availability**	Environmental change	Facilitation of healthy behaviour	Offering fruit and vegetables
**Costs**	Environmental change	Facilitation of healthy behaviour	Offering fruit and vegetables
**Intention**	Self regulation	Teach skills	Incompany mindfulnesstraining with exercises aimed enacting on intentions

Table 4 Methods and strategies selected for increasing work engagement

Individual determinant	Theoretical method	Practical strategy	Tools and Materials
**Perceived behavioural control**	Self regulation	Teach skills	Incompany Mindfulness training with goals, barriers and actions exercises
	Goal setting	Facilitate formulation of goals and reflection	E-coaching: Employees formulate goals and reflect in personal e-diary on website. Coach reads and stimulates reflection
	Reinforcement	Providing positive feedback	E-coaching: coach provides positive feedback
**Organisational based self-esteem**	Goal setting	Facilitate formulation of goals and reflection	E-coaching: E formulate goals and reflect in personal e-diary on website. Coach reads and stimulates reflection
	Reinforcement	Providing positive feedback	E-coaching: coach provides positive feedback
**Optimism**	Self regulation	Teach skills	Incompany Mindfulness training with exercises aimed at optimism
**Perceived barriers**	Self regulation	Teach skills	Incompany Mindfulness training with exercices aimed at perceiving and reperceiving barriers of work engagement
	Goal setting	Facilitate formulation of goals and reflection	E-coaching: E formulate goals and reflect in personal e-diary on website. Coach reads and stimulates reflection.
	Reinforcement	Provide positive feedback	E-coaching: coach provides positive feedback
**Social support**	Mobilising social support	Form groups	Group or duo discussions in incompany mindfulness sessions to discuss

			exercises. Finding a buddy is stimulated during the sessions.

2.1.4 Step 4: Program Development (November – December 2009)

The detailed interpretation of programme strategies and materials has been tested on intended implementers and recipients by means of two participatory focus group discussions. Key figures as well as employees were invited to these focus group discussions. Details of tools and materials have been discussed and filled in by the target group during these sessions as they have insight into the possibilities and impeding factors of the organisation. Besides tools and materials, compliance and participation have been discussed.

The intervention mapping process has so far been completed until step 4. Currently (December 2009/ January 2010) step 5 is being completed. Step 5 and 6 will be completed from January 2010 until February 2011.

2.1.5 Step 5: Adoption and implementation (January 2010 – January 2011)

In this step, the focus will be on adoption and implementation (including consideration of program sustainability). The product for step 5 will be a detailed plan for accomplishing program adoption and implementation by influencing behaviour of individuals who will make decisions about adopting and using the program.

2.1.6 Step 6: Evaluation

Step 6 of the intervention mapping protocol will take place in phase 2 of this study.

2.2 Phase 2: Evaluation

The effects of the intervention will be evaluated by a randomised controlled trial (RCT) with two arms. Employees in the intervention group will receive the newly developed program in addition to the current health promotion activities (eg. Incompany fitness). The control group will 'only' receive the latter. Measurements will take place for both groups at the same time: at baseline (T0), after 6 months (T1) and after 12 months (T2).

2.3 Intervention

The intervention consists of a mindfulness based coaching program, i.e. a program of mindfulness sessions and a E-coaching program. Additionally supporting tools such as fruit, a buddy-system and routes for lunch walking will be offered to the participants in the intervention group.

2.3.1 Mindfulness

Mindfulness can be described as “an open, undivided observation of what is occurring both internally and externally” (Brown & Ryan, 2003, p823). Mindfulness is a moderator on the intention-behaviour relationship, such that intentions predict behaviour among mindful individuals (Chatzisarantis & Hagger, 2007, p 668, p671). Counterintentional habits and thoughts have less effect on mindfulness individuals (Chatzisarantis & Hagger, 2007, p669) probably because they are more aware of their behaviour and have a greater sense of control and efficacy (Chatzisarantis & Hagger, 2007, p669). In other words: non-mindful persons have a lower chance to carry out their intentions because they lack control and efficacy.

Mindfulness training can be seen as a strategy of self regulation (Chatzisarantis & Hagger, 2007; Brown and Ryan, 2003; Tapper et al, 2009; Baer, 2009). Of all self regulation strategies, it hypothetically fits the target group best as it is likely to fit individuals who have a high need for cognition because they pay attention to relevant arguments, according to Chatzisarantis & Hagger (2007). Implementation intentions for example, has been proven to be an effective strategy, but as it focuses on automatic processing, it fits people with a low need for cognition, who prefer using heuristic rules (Chatzisarantis & Hagger, 2007). As the target group mainly consists of highly educated employees (researchers), it is likely that they have a high need for cognition.

Mindfulness has shown to be effective in improving several aspects of both mental and physical health (Grossman, Niemann, Schmidt & Walach, 2004) . It has been proven to be effective in interventions to increase physical activity (Chatzisarantis & Hagger, 2007; Tapper, Shaw, Ilsley, Hill, Bond & Moore, 2009) and to stimulate weight reduction (Tapper et al, 2009). Mindfulness can be related to the contruct of work engagement (see introduction: absorption, vigour and dedication, Schaufeli et al, 2002). Mindful attention to present

activities (Baer, 2003) can be related to absorption. Subjective well-being as a result of mindfulness (Brown & Ryan, 2003) is related to vigour- although this is more narrow definition. Mindful attention implicits involvement (Langer & Moldoveanu, 2000), which is needed for dedication (Schaufeli et al, 2001). Also, mindfulness based intervention has proven to reduce work stress (Nyklicek& Kuijpers, 2008). In this organisation, stress is mainly caused by workload and organisational change, according to the interviewees. Workload and organisational change are important – but difficult to change or even unchangeable - determinants of work engagement (Xanthopoulu, Bakker, Demerouti & Schaufeli, 2008).

The mindfulness training consists of mental exercises (metaphors) used in other effective lifestyle interventions (among others Tapper et al, 2009) to improve dietary behaviour and physical activity and to reduce sedentary behaviour. It also consists of exercises aimed at work engagement. The duration of the whole training is 8 weeks, as this the duration which is needed to learn a new way of thinking (Baer, 2003). One session in this study will take one and half hours, which is shorter then the usual duration (2,5 hours) but in-company mindfulness training of shorter duration have also proven to have an effect on the state of mindfulness (Klatt, Buckworth & Malarkey, 2008). The training will be held during the same hours as in-company fitness is available. Locations are available at the worksite. Group size will be about 15 employees. Employees will receive an invitation for the first meeting but will be able to change hours if necessary. During the sessions, there will be instruction for homework exercises. Mindfulness training will be given by certified trainers.

2.3.2 E-coaching

Employees will receive coaching by email, to monitor, reflect on and achieve set goals for physical activity and dietary behaviour and work engagement. Employees monitor, set goals and reflect in a web-based journal, to which only the coach has access (besides the employee). Coaching by email has been proven to be effective to reduce weight loss (Van Wier, Ariëns, Deckers, Hendriksen, Pronk, Smid & van Mechelen, 2006). Coaching will be done by the mindfulness trainers who also give the training and will implement the mindfulness principles learned in the training. Coaching will follow after the 8 weeks of mindfulness training. Employees and key-figures indicated in focus groups that they reckoned a high frequency of coaching in the beginning and a lower frequency as the program continues to be most beneficiary. If employees encounter problems (mentally or physically), coaches will refer them to the occupational physician or the company social worker. If necessary, the coach may suggest some options in the current offer of the organisation (such as the possibility of in-company fitness or a training by Bureau talent).

2.3.3 Supporting elements

There are three supporting elements in this intervention. The first one is offering fruit. A piece of free fruit will be offered at the incompany mindfulness training and can be collected at the silence centre on the premises of the organisation. The second supporting element is providing routes for lunch walking in the nearby forest. These routes may provide information on the surroundings, nature, etc. Integrated in the routes is a stop at the local greengrocer. The third supporting element is the stimulating of finding a buddy for several purposes: to attend to mindfulness sessions, lunch walking, exercising and commuting to work by bike. Participants will be stimulated to find a buddy by the mindfulness trainer and later on by the coach. During the mindfulness sessions, homework exercises will be discussed with a buddy.

3. Study Population

3.1 Population

The study population consists of employees of the RIVM. The number of employees is approximately 2000. The study population consists of mainly highly educated employees. Most of the employees are office workers. Mean age of the employees is 43 years.

3.2 In- and exclusion criteria

All employees of the RIVM will have the possibility to participate in this study, in case they:

- are not taking sick leave for more than 4 weeks at the moment of invitation
- have signed an informed consent form.

3.3 Sample size calculation

The sample size was based on finding an effect on physical activity, measured by the SQUASH questionnaire (Wendel-Vos et al, 2003). The baseline mean score of minutes of all activities per week (of all intensities) is assumed to be 3045 (SD=931) according to Wendel-Vos et al (2003). Based on the effects of lifestyle interventions on other behavioural and health measures, an effect of 10% is expected to be relevant and feasible. This means a

score of 3349,5 minutes of activity per week in the intervention group versus 3045 minutes of activity per week in the control group. To demonstrate the difference between both groups with a power of 90% and a two sided alpha of 5%, both groups need to consist of 197 employees. This number is also sufficient to demonstrate an effect in work engagement (measured with the UBES, Schaufeli & Bakker 2003): the mean score for work engagement is assumed to be 3,82 (on scale from 0 to 6, SD=1,1), an effect of 10% means a score of 4,20 in the intervention group, versus 3,82 in the intervention group.

Accounting for a loss to follow-up of 25% in twelve months, each group needs to consist of 263 employees at baseline, summing up to 526 employees for the two groups.

Based on the source population of 2000 employees, the number of 526 participants is reached at a participation rate of 26,3%. This percentage is considered well feasible as all employees will be invited to participate.

4. Methods

4.1 Recruitment

Employees will be invited to participate by means of a letter of invitation, which will be distributed by their supervisor. Supervisors will receive packages (envelopes) for the employees. Each package contains information for participants (see appendix 1) an invitation letter for the employee (see appendix 2), a reply card, which is a registration and informed consent (see appendix 3) and a reply envelope. These packages will be accompanied by a letter for the supervisor (see appendix 4). Supervisors will be asked (not obliged) to send an email to let the employees know they have received an invitation.

Before the actual invitation will be sent, “VIP in Onderzoek” will be promoted (see also 7.2) using existing communication channels in collaboration with the organisation’s HRM and communication department (for example intranet, posters, newsletters and RIVM@home, the personnel magazine). After the official invitation, actions to remind employees will be taken. Reminders will be placed on the intranet (“actueeltjes”) and a (not personalised) email will be sent to all employees.

When the participant has completed and returned the answering card and informed consent form, they will receive an invitation for the first measurement. Measurement of body mass index (BMI) BMI and waist circumference will be performed by the researcher and/or the research assistant. These measurements will take place on the premises of the

organisation. A subsample will be invited to wear an accelerometer (see 4.3.1.1). Questionnaires will be handed out during this measurement. Questionnaires can be handed in blinded boxes at the entrance of the property of the organisation (next to the safety guards) or with the researcher/ research assistant.

4.2 Randomisation

Randomisation will be conducted at individual level. The randomisation will be performed directly after baseline measurement (T0) and will be prepared and performed by an independent researcher. A computer-generated randomisation will be examined by using SPSS. As a result of the nature of the intervention, blinding the participants is not possible.

4.3. Measurements

4.3.1 Primary endpoints

Primary endpoints of this study are energy balance related behaviours and work engagement.

4.3.1.1 Energy balance related behaviours

Physical activity will be assessed by the Short Questionnaire to Assess Health Enhancing Physical Activity (SQUASH) (Wendel-Vos et al, 2003). It measures duration, frequency and intensity of work transportation, household activities, leisure activities and work activities.

Questions related to **sedentary behaviour at work** will include the time spent (in minutes per day) on 1) computer use, 2) reading, 3) meetings, 4) calling and 5) other activities.

Additionally, physical activity and sedentary behaviour will be measured objectively in a random sample of 200 participants of both the intervention (n=100) and control group (n=100). This subsample will be asked to wear the accelerometer during a period of 7 days. Participants will be invited to wear an accelerometer at the measurement moment. One week afterwards, they will be asked to hand it in at the researcher/research assistant on appointment.

Dietary behaviour will be assessed by means of the fruit and vegetable intake. This will be done using the Short Fruit and Vegetable Questionnaire (Van Assema et al, 2002). The questionnaire consists of 10 questions: 6 about fruit consumption and 4 about consumption of vegetables.

4.3.1.2 Work engagement

The Utrecht Work Engagement Scale (UWES), will be used to measure work engagement. The UWES consist of three aspects: vigour (6 items), dedication (5 items), and absorption (6 items) (Schaufeli & Bakker, 2003; 2004; Schaufeli et al, 2002).

4.3.2 Secondary endpoints

Mental health
Mental health will be assessed using the mental health items from the RAND-36 using the validated Dutch version (Van der Zee & Sanderman, 1993). Participants will be asked to indicate for five items on a six point scale how often they felt anxious, depressed, calm, sad and happy during the past four weeks.

Vitality
Vitality is measured by means of the 4 vitality items from the RAND-36 using the validated Dutch version (Van der Zee & Sanderman, 1993). Participants will be asked to indicate on a six point scale how often they felt full of life, worn out, tired and full of energy during the past four weeks.

General health perceptions
General health perceptions are measured by means of the 5 items from the RAND-36 using the validated Ducth version (Van der Zee & Sanderman, 1993). Participants will be asked to indicate how they perceive their health (on a five point scale from excellent to bad) and to indicate on four propositions to which extent they agree on a five point scale.

BMI and waist circumference
Data of body weight and body height will be measured to calculate BMI. Height will be measured to the nearest 0.5 cm without shoes. Weight will be measured to the nearest 0,1 kg in participants wearing indoor clothing and no shoes, after emptying their pockets.

Waist circumference will be measured by the researcher and/or research assistant and is measured as midway between the lower rib margin and the iliac crest to the nearest 0.1 cm (Lean, Han & Morrison, 1995). Participants are measured standing up, breathing out gently, wearing indoor clothing, after emptying their pockets.
Height, weight and waist circumference measurements will be performed by the researcher and/or research assistant on the property of the organisation. The researcher and research assistant will be trained in measuring waist circumference in a correct way. Measurements take place on the premises of the organisation (see also 4.1 for procedure).

Absenteeism and presenteeism
Sick leave will be determined using the World Health Organisation Health and Work Performance Questionnaire (WHO-HPQ) measuring loss of productivity due to decreased performance while at work (presenteeism) and sick leave (absenteeism) (Kessler et al, 2003; Kessler et al 2004). Furthermore, sick leave data will be collected from the company's registrations.

Need for recovery
The 11-item ‘need for recovery scale’ from the Dutch version of the Questionnaire on the Experience and Evaluation of Work (Dutch abbreviation VBBA) will be used. The VBBA has been evaluated among 601 workers from various organisation in the Netherlands, and has showed satisfactory reliability. The ‘need for recovery scale’ has shown to be adequate and reliable (Van Veldhoven & Broersen, 2003).

4.3.3 Other endpoints

Mindfulness
The level of mindfulness will be measured using the Dutch version of the validated Mindful Attention Awareness Scale (MAAS) (Brown & Ryan, 2003; Schroevers, Nyklicek & Topman, 2008). This 15 item scale measures on a 6 point scale the frequency of everyday mindfulness experiences.

Determinants of behaviour and behaviour change
As the aim of this study is to improve EBRB, it is necessary to measure its determinants. Based on models of behaviour and behavioural change, questions are asked on perceived behavioural control, intention and perceived barriers (lack of time) for physical activity and

dietary behaviours (De Vries, Kremers, Smeets, Brug & Eijmael, 2008; McEachan, Lawton, Jackson, Conner& Lunt, 2008).

Need for cognition

The need for cognition will be assessed using the short version of the Need for Cognition Scale (Cacioppo, Petty, Feinstein, & Jarvis, 1996). This scale consists of 18 items on which participants have to indicate on five point scale to which extent it is characteristic for them. Items assess engagement in and enjoyment of thinking, learning and intellectual cognitive activities.

4.3.4 Data analysis

4.3.4.1 Statistical analysis

The effectiveness of the intervention will be analysed by means of a regression analysis (analysis of covariance) with the outcome measure at follow-up (T1: 6 months and T2: 12 months) as the dependent variable and adjusting for the baseline levels of the outcome measure. In addition, a Generalised Estimated Equations (GEE) analysis will be performed for the long term effects (T0- T2). All statistical analyses will be performed according to the intention-to-treat principle. For all analyses a two tailed significance level of <0.05 is considered statistically significant. The regression analyses and GEE will be performed with SPSS 16.0 (SPSS Inc. Chicago, Illinois, USA).

4.3.4.2 Economic evaluation

4.3.4.2 Economic evaluation

This project will include an economic evaluation that will be performed by a PhD-student, Ms. H. van Dongen, as part of her PhD-thesis. The economic evaluation will consist of a cost-effectiveness analysis (CEA) and a cost-benefit analysis (CBA). The aim of the CEA will be to determine and compare the difference in total costs between the intervention and the control group, and to relate these differences in costs to the difference in effects between the two groups. The primary outcome measures of the economic evaluation are physical activity (total score on the SQUASH) and work engagement (UWES), as described under the primary endpoints. The aim of the CBA will be to compare the costs with obtained benefits. Both analyses will be performed from a societal perspective and from a company

perspective. The time horizon for all analyses will be 12 months, similar to the trial. Analyses will be performed according to the intention-to-treat principle.

Cost-analysis

From a societal perspective, all relevant intervention costs, health care costs, and production losses will be included. From a company perspective, only costs and outcomes relevant to the company will be included. Health care costs and production losses will be valued using the guidelines published in the updated handbook for economic evaluation in the Netherlands, issued by the Dutch Health Care Insurance Board.

The following costs will be collected in this study:

- Intervention costs: the costs of the development and implementation of the intervention. Development costs consist of the cost of time spent by the researchers and contractors (e.g. graphic designer, web developer) on the development of the intervention and its accompanying website. These will be calculated on the basis of estimations of time investments, and based on invoices from contractors. Implementation costs consist of the costs for website hosting and maintenance, costs of group meetings and e-mail contacts with the Mindfulness coach. Contacts with the coaches (frequency and duration of contacts) will be recorded by the coaches.
- Health care costs: the costs of general practice care, physiotherapy, other health care providers, prescriptions of medication and hospitalisation. These data will be collected through prospective cost diaries filled in on a three monthly basis during the entire follow-up period.
- Costs due to loss of production: the costs of absenteeism and presenteeism. These will be measured with the Dutch version of the WHO-HPQ (see secondary endpoints). Absenteeism will also be derived from the company registrations. Participants will be asked to complete the WHO-HPQ once every month.

Multiple Imputation (MI) using the Multivariate Imputation by Chained Equations (MICE) algorithm will be used to impute missing cost and effect data. Mean differences in costs between the study groups will be estimated. Confidence intervals (95%) around these mean differences will be obtained by bias corrected and accelerated (Bca) bootstrapping.
For the CEA, cost-effectiveness ratios will be calculated by dividing the difference in the mean costs between the study groups by the difference in the mean effects between the study groups. The uncertainty surrounding the cost-effectiveness ratios will be estimated using bootstrapping techniques. The bootstrapped cost-effect pairs will be used to estimate cost-effectiveness planes and cost-effectiveness acceptability curves. Furthermore,

sensitivity analyses of the most important cost drivers will be performed to assess the robustness of the results. For the CBA , net benefit will be estimated.

5. Study procedures

The control group as well as the intervention will be asked to complete baseline and two follow up measurements. A special VIP page will be created on the intranet for all participants, summing up the links of the current offer which are related to EBRB and work engagement. These links are already on the intranet in other tabs. A login and password will be provided only once.

Only employees assigned to the intervention group will participate in mindfulness training of 8 weeks, followed by e-coaching. Employees in the control group will only receive access to the special VIP page.

5.1 Withdrawal of individual subjects

5.1.1 Specific criteria for withdrawal

None

5.1.2 Replacement of individual subjects after withdrawal

None

5.1.3 Follow-up of subjects withdrawn from treatment

None

5.1.4 Premature termination of the study

As this RCT is non-medical, but stimulates healthy behaviour instead, the study is not likely to be terminated prematurely.

6 Safety reporting

6.1 Section 10 WMO event

In this questionnaire study, no interventions will be applied including investigational medicinal research.

6.2 Adverse and serious adverse events

This is not an investigational medicinal research

6.3 Follow-up of adverse events

No serious adverse events from the questionnaires and program are expected, for this reason no follow-up of adverse events will be performed. The program consists of mindfulness sessions and e-coaching to improve some aspects of physical activity, dietary behaviour and work engagement. If employees encounter problems (mentally or physically) at any moment during the program, trainers and coaches will refer them to the occupational physician or the company social worker, physically present on the premises of the organisation. Agreements on when to refer to what and who will be made between the trainers and coaches and the occupational physician, company social worker and the head of bureau Talent. To the opinion of the researchers no insurance will be needed and therefore, the researchers ask for dispensation for the insurance.

6.4 Data Safety Monitoring Board (DSMB)

Not necessary.

7. Ethical considerations

7.1 Regulation statement

The study will be conducted according to the principles of the Declaration of Helsinki.

7.2 Recruitment and consent

The usual and available communication channels of the RIVM will be used in order to recruit participants. Usual and available communication channels are intranet, internet, the personnel magazine (RIVM@Home), posters on bulletin boards and digital newsletters. Efforts will be made to create support amongst supervisors and directors (see also 4.1). Consenting participants will be assigned to one of the two groups. Although contamination between co-workers within one department is possible, chances of this happening are being estimated low as they both get access to a special tab or hyperlink on the intranet. The employees are therefore being assigned to either the intervention group or the control group on an individual basis. An informed consent is included (see appendix 4).

7.3 Benefits and burden assessment

Benefits for the participants are:
- Intervention group: participation in a programme consisting of mindfulness sessions, followed by e-coaching, with supporting elements as providing fruit
- Intervention group: if the intervention proves to be effective: improvement in EBRB and work engagement
- Control group: promotion of the existing offer (such as training, fitness and self tests) through the special VIP page on the intranet and participation in those activities.

The burdens for the participants (both control group and intervention group) are:

- Completing three questionnaires during the study period of one year
- Completing a very small questionnaire every 3 months (WHO-HPQ)

The completion of one questionnaire will take about 40 minutes. However, the burdens for the participants are negligible when compared to the profits.

7.4 Compensation for injury

In this questionnaire study there are no risks for individual participants. The intervention consists of mindfulness sessions and e-coaching to increase vitality by improving some

aspects of physical activity, dietary behaviour and work engagement. To the opinion of the researchers no insurance will be needed and therefore, the researchers ask for dispensation for the insurance.

7.5 Compliance and incentives

To enlarge compliance, employees and key figures were asked in 2 focus group discussions about suitable incentives. Most suitable was considered a voucher for an in-company chair massage session. This will be offered to every 10th participant returning the questionnaire. All participants will be offered little VIP gadgets, such as a pen or a sticker. Employees and key figures believed that participants' drivers were principally of societal character, such as participating in this RCT to help science. Compliance could furthermore be enlarged by organising special clinics, master classes and trials of sports (for example yoga) and by communicating appealingly to their values, that this study has a societal impact. The special VIP tab or hyperlink on the intranet provides information on this subject.

To optimise compliance to the sessions, the e-coach and the questionnaires, participants will be contacted pro-actively. For example, if a participant doesn't attend to a mindfulness session for which he or she has signed up for, the participant will receive an email or a phone call by the researcher/ research assistant to let him or her know it was noticed and will be positively stimulated to attend to the next session. The same applies to the questionnaire. The e-coach will pro-actively contact the participant if he or she hasn't used the e-log-book.

8. Administrative aspects and publication

8.1 Handling and storage of data and documents

8.1.2 Data assessment

Questionnaires will be assessed by hard copy.

8.1.3 Data entrance

Data obtained from the hard copy versions will be entered by hand into a coded SPSS file using data entry (version 15.0 for Windows). The data entrance will be performed by the research assistant. The inserted data will be double checked in 20% of the data.

8.1.4 Data storage

Person specific codes will be stored in an Excel database, secured by a code, and will only be accessible to the principal researcher, the research assistant, and a member of the Ondersteunend Beheer Personeel (OBP). Furthermore, the file that links the personal information of the workers (i.e. work e-mail addresses, names and/ or workplace information) and the person specific codes will be stored at the restricted network drive by and will be only accessible to the principal researcher and research assistant. Data obtained from the questionnaires will be stored in a SPSS database by using the person specific codes. The SPSS database will be secured by a code and will be only accessible to the principal researcher, the research assistant and a member of the OBP.

8.2 Amendments

All substantial amendments will be notified to the METC and to the competent authority. Non-substantial amendments will not be notified to the accredited METC, but will be recorded and filed.

8.3 Annual progress report

The METC will be notified in the case of serious adverse events/ serious adverse reactions, other problems, and amendments.

8.4 End of study report

The investigator will notify the accredited METC of the end of the four year study within a period of 8 weeks. The investigator will send a final report with the results of the study, including any publications/abstracts of the study, to the accredited METC.

References

Bartholomew, L.K., Parcel, G.S., Kok, G., Gottlieb, N.H., (2006) *Planning Health Promotion Programs: An Intervention Mapping Approach*. San Francisco, CA: Jossey-Bass

Bravata, D.M., Smith-Spangler, C., Sundaram, V. et al. (2007) Using Pedometers to Increase Physical Activity and Improve Health: A Systematic Review. *JAMA: The Journal of the American Medical Association 298*(19):p2296-2304.

Brown, K.W.& Ryan, R.M (2003) The Benefits of Being Present: Mindfulness and Its Role in Psychological Well-Being *Journal of Personality and Social Psychology, 84(4)* 822-848

Cacioppo, J. T., Petty, R. E., Feinstein, J, A., & Jarvis, W. B. G. (1996). Dispositional differences in cognitive motivation: The life and times of individuals varying in need for cognition. *Psychological Bulletin, 119,* 197–253.

De Vries H, Kremers SP, Smeets T, Brug J, Eijmael K. (2008)The effectiveness of tailored feedback and action plans in an intervention addressing multiple health behaviours. *American Journal of Health Promotion*,*22*:417-25

Demerouti, E., Bakker, A.B. (2006), "Employee well-being and job performance: where we stand and where we should go", in Houdmont, J., McIntyre, S. (Eds),*Occupational Health Psychology: European Perspectives on Research, Education and Practice*, ISMAI Publications, Maia, Vol. Vol. 1.

Dugdill, L., Brettle, A., Hulme,C., McCluskey. C., Long, A.F. (2008) Workplace physical activity interventions: a systematic review. *International Journal of Workplace Health Management Vol. 1 No. 1,* p20-40.

Van Duijvenbode, D.C., Hoozemans, M.J.M., Van Poppel, M.N.M & Proper, K.I. (2009) The relationship between overweight and obesity, and sick leave: a systematic review. *International Journal of Obesity, 33:* p807-816

Engbers, L.H., van Poppel, M.N., Chin A Paw, M.J., van Mechelen W. (2005) Worksite health promotion programs with environmental changes: a systematic review. *American Journal of Preventive Medicine.;29*(1):61

Fine J.T., Colditz G.A., Coakley E.H., Moseley G., Manson J.E., Willett W.C., and Kawachi I. (1999). A Prospective Study of Weight Change and Health-Related Quality of Life in Women. *JAMA: The Journal of the American Medical Association* 282 (22): 2136-2142.

González-Roma, V., Schaufeli, W.B., Bakker, A.B., & Lloret, S. (2006). Burnout and engagement: Independent factors or opposite poles? *Journal of Vocational Behaviour*. 68, 165–174.

Hill, J.O. (2006) Understanding and addressing the epidemic of obesity: an energy balance perspective. *Endocrine Reviews: 27,* p750–761

Hertz, R.P., Unger, A.N., MCDonald, M., Lustik M.B. & Biddulph-Krentar J. (2004) The impact of obesity on work limitations and cardiovascular risk factors in the U.S. workforce. *Journal of Occupational and Environmental Medicine: 46*, p1196-1203.

Hoeymans, N., Lindert, H., & Westert, G.P. (2005). The health status of the Dutch population as assessed by the EQ-6D. *Quality of Life Research*, *3*, 655-663

Jansen, N.W.J., Kant, IJ, van den Brandt, P.A, (2002) Need for Recovery in the Working, *International Journal of Behavioural medicine 9(4),* 322–340

Kaukua J, Pekkarinen T, Sane T, Mustajoki P (2002): Health-related quality of life in WHO class II – III obese men loosing weight with very-low-energy diet and behaviour modification: A randomized clinical tria**l.** *International Journal of Obesity Related Metabolic Disorders 26***:**487-495.

Kaukua J, Pekkarinen T, Sane T, Mustajoki P (2003): Health-related quality of life in obese outpatients losing weight with very-lowenergydiet and behaviour modification – A 2-y follow-up study. *International Journal of Obesity 27***:**1233-1241.

Kessler,R.C., Ames,M., Hymel,P.A., Loeppke,R., McKenas,D.K., Richling,D.E., Stang,P.E.& Ustun,T.B. (2004) Using the World Health Organization Health and Work Performance Questionnaire (HPQ) to evaluate the indirect workplace costs of illness. *Journal of Occupational and Environmental Medicine 46*

Kessler,R.C., Barber,C., Beck,A., Berglund,P., Cleary,P.D., McKenas,D., Pronk,N., Simon,G., Stang,P., Ustun,T.B. & Wang,P. (2003) The World Health Organization Health and Work

Performance Questionnaire (HPQ). *Journal of Occupational and Environmental Medicine 45:*156-174

Kim, D. & Kawachi I. (2008). Obesity and Health-Related Quality of Life., 234-260. Oxford University Press, USA.

Langer, E. J. & Moldoveanu, M. (2000) The construct of mindfulness. *Journal of Social Issues 56* 1–9.

Lean, M.E., Han, T.S. & Morrison, C.E. (1995) Waist circumference as a measure for indicating need for weight management, *Britisch Medical Journal,311*, p158-161

Lemmens, V.E.P.P., Oenema, A., Klepp, K.I., Hendriksen, H.B. & Brug, J. (2008) A systematic review of the evidence regarding efficacy of obesity prevention interventions among adults. *Obesity reviews, 9*, p446-455

Lombard, C.B., Deeks, A. & Teede, H.J. (2009) A systematic review of interventions aimed at the prevention of weight gain in adults. *Public Health Nutrition, 12(11),* p2236–2246

Lyubomirsky, S., King, L. A., & Diener, E. (2005). The benefits of frequent positive affect: Does happiness lead to success? *Psychological Bulletin, 131*, 803-855.

McEachan, R.C., Lawton, R.J. Jackson, C., Conner, M. & Lunt, J.(2008).Evidence, Theory and Context: Using intervention mapping to develop a worksite physical activity intervention. *BMC Public Health*, *8*:326-38.

McHorney,C.A., Ware,J.E., Jr., Raczek,, A.E. (1993) The MOS 36-Item Short-Form Health Survey (SF-36): II. Psychometric and clinical tests of validity in measuring physical and mental health constructs. *Medical Care* 31:247-263

Neovius. K., Johansson, K., Kark, M. & Neovius, M. (2009) Obesity status and sick leave: a systematic review. *Obesity reviews 10,* p17-27

Nyklïcek, I., & Kuijpers, K. F. (2008). Effects of mindfulness-based stress reduction intervention on psychological well-being and quality of life: Is increased mindfulness indeed the mechanism? *Annals of Behavioural Medicine,35*, 331–340

Pronk, N.P., Martinson, B., Kessler, R.C., Beck, A.L., Simon, G.E. & Wang O. (2004) The association between work performance and physical activity, cardiorespiratory fitness, and obesity. *Journal Occupational Environmental Medicine:46*,p19-25.

Proper, K.I., Hildebrandt, V.H.(2007) Sitting time and socio-economic differences in overweight and obesity. *International Journal of Obesity, 31*, p169-176

Proper, K.I., Hildebrandt, V.H.(2009) Overweight and obesity among Dutch workers: differences between occupational groups and sectors. *International Archives of Occupational and Environmental Health* [Epub ahead of print].

Rippe JM, Price JM, Hess SA, Kline G, DeMers KA, Damitz S, Kreidieh, I.& Freedson, P (1998) Improved psychological well-being, quality of life, and health practices in moderately overweight women participating in a 12-week structured weight loss program. *Obesity Research, 6*: 208-18.

Ross, M.K., Milsom, V.A., Rickel, K.A., DeBraganza, N., Gibbons L.M., Murawski, M.E. & Perri, M.G. (2009) The contributions of weight loss and increased physical fitness to improvements in health-related quality of life, *Eating Behaviours* :10; 84–88

Schaufeli ,W.B., Bakker, A.B.& Van Rhenen W. (2009). How changes in job demands and resources predict burnout, work engagement, and sickness absenteeism. *Journal of Organizational Behaviour*.

Schaufeli, W.B.& Bakker, A.B. (2004). Job demands, job resources, and their relationship with burnout and engagement: a multi-sample study. *Journal of Organizational Behaviour* 25 (3): 293-315.

Schaufeli, W.B., Salanova, M., Gonzalez-roma, V.& Bakker AB. (2002). The measurement of engagement and burnout: A two sample confirmatory factor analytic approach. *Journal of Happiness Studies* 3 (1): 71-92.

Seidell, J.C. (1998) Societal and personal costs of obesity. *Experimental and Clinical Endocrinology & Diabetes*; *106* : 7–9 (supl 2)

Sluiter, J. K., Van der Beek, A. J., & Frings-Dresen, M. H. W. (1999). The influence of work

characteristics on the need for recovery and experienced health: A study on coach drivers. *Ergonomics, 42,* 573–583.

Van der Zee KI, Sanderman R.(1993) *Het meten van de algemene gezondheidstoestand met de RAND-36. Een handleiding.* Groningen: noordelijk centrum voor gezondheidsvraagstukken

Van Veldhoven, M. & Broersen, S. (2003) Measurement quality and validity of the "need for recovery scale", *Occupational and Environmental Medicine; 60,*3-9 (supl 1)

Visscher TLS, Seidell JC (2004). Time trends (1993–1997) and seasonal variation in body mass index and waist circumference in the Netherlands. *International Journal of Obesity Related Metabolic Disorders, 28:* 1309–1316.

Wendel-Vos,G.C., Schuit, A.J., Saris,W.H.& Kromhout,D (2003) Reproducibility and relative validity of the short questionnaire to assess health-enhancing physical activity. *Journal of Clinical Epidemiology 56*:1163-1169

World Health Organization (WHO) (2004) Obesity: Preventing and Managing the Global Epidemic. *Report of a WHO Consultation*. World Health Organization: Geneva.

Xanthopoulou, D., Bakker, A.B., Demerouti, E.& Schaufeli, W.B. (2009). Work engagement and financial returns: A diary study on the role of job and personal resources. *Journal of Occupational and Organizational Psychology* 82 (1): 183-200.

Xanthopoulu, D., Bakker, A.B., Demerouti, E. & Schaufeli, W.B. (2008) Reciprocal relationship between job resources, personal resources and work engagement, *Journal of Vocational Behaviour, 74,* 235-244

Appendices

1. Participant Information
2. Participant letter of invitation
3. Supervisors accompanying letter
4. Participant registration form/ Informed consent
5. Costs diary
6. Questionnaire

Appendix 1: Participant information

VIP

VITAAL IN PRAKTIJK

VIP in onderzoek

WERKEN BIJ HET RIVM IS LEUK EN VOORAL UITDAGEND. ONZE HERSENEN WERKEN OP VOLLE TOEREN TERWIJL WE LICHAMELIJK STEEDS INACTIEVER WORDEN. PRIVÉ HEBBEN WE OOK VAAK ONZE HANDEN VOL. DAAROM IS HET SOMS MOEILIJK OM VOLDOENDE TE BEWEGEN EN GEZOND TE ETEN. DAARNAAST IS HET BELANGRIJK DAT JE WERK JE MEER ENERGIE GEEFT DAN DAT HET KOST. VITAAL IN PRAKTIJK (VIP) RICHT ZICH OP DE OPTIMALISATIE VAN DE GEZONDHEID VAN DE MEDEWERKER. DAARTOE WORDEN PROGRAMMA'S ONTWIKKELD EN GEËVALUEERD MIDDELS ONDERZOEK BINNEN BEDRIJVEN EN ORGANISATIES .

VIP in onderzoek

VIP project

Het VU medisch centrum (VUmc) en TNO hebben samen met RIVM een VIP programma op maat gemaakt voor RIVM medewerkers. Bij de ontwikkeling van dit programma is de behoefte onder RIVM medewerkers gepeild en de mening gevraagd ten aanzien van de invulling van het programma. Een passend programma is het resultaat.

DOEL

Met het VIP project bij het RIVM wordt inzicht verkregen in de effectiviteit van een programma op maat. Hierbij is het behoud en de bevordering van een gezonde leefstijl en bevlogenheid de doelstelling van dit programma.

VOOR WIE IS DIT ONDERZOEK?

Iedereen die werkzaam is bij het RIVM kan meedoen. Zodra u het toestemmingsformulier ondertekend heeft opgestuurd, wordt u uitgenodigd voor verdere deelname.

WAT HOUDT HET ONDERZOEK IN?

Wij vragen u op verschillende momenten een vragenlijst in te vullen. De momenten zijn:

- voor de start van het programma, begin 2010
- 6 maanden na de start, medio 2010
- 12 maanden na de start, begin 2011

WAT BETEKENT MEEDOEN VOOR U?

Het levert u inzicht op in uw leefstijl, bevlogenheid en vitaliteit. Daarnaast krijgt u de mogelijkheid om door middel van het VIP programma met deze inzichten aan de slag te gaan zodat u optimaal gezond en bevlogen blijft.

HOE WERKT HET?

Om het onderzoek goed uit te kunnen voeren testen wij twee verschillende programma's. Nadat we het toestemmingsformulier hebben ontvangen, wordt u willekeurig ingedeeld bij een van de programma's.

VERTROUWELIJKHEID VAN DE GEGEVENS

Alle gegevens worden vertrouwelijk behandeld en anoniem verwerkt. Gegevens worden alleen gebruikt voor wetenschappelijke doeleinden. Uw persoonlijke gegevens worden niet aan derden (zoals uw leidinggevende) verstrekt. De onderzoeksgegevens worden gerapporteerd op groepsniveau: u kunt dus nooit persoonlijk herkend worden.

VRIJWILLIGHEID

Deelname aan dit project is op vrijwillige basis. Wij hopen dat u enthousiast bent en blijft! Uiteraard hebt u de vrijheid om deelname om voor u relevante redenen tussentijds te beëindigen. Wij hopen natuurlijk dat u wilt blijven deelnemen gedurende de hele periode van het project.

ZIJN ER RISICO'S VERBONDEN AAN HET ONDERZOEK?

De medisch ethische toetsingscommissie van het VUmc heeft toestemming gegeven voor dit project. Er zijn geen risico's verbonden aan uw deelname.

Het onderzoeksteam

Jantien van Berkel *Projectmedewerker*
Dr. Cécile Boot *Begeleider*
Dr. Karin Proper *Projectleider*
Prof. Dr. Allard van der Beek *Hoogleraar Arbeidsepidemiologie*
Prof. Dr. Ir. Paulien Bongers *Bijzonder hoogleraar Preventie van arbeidsgerelateerde klachten*

Meer informatie?

Hebt u vragen of opmerkingen over het onderzoek, neemt u dan gerust contact op met VIP via vipinonderzoek@vitaalinpraktijk.nl of 06-22002759.

VIP is een project dat geïnitieerd is door Delta Lloyd / OHRA Zorgverzekeringen en uitgevoerd wordt door Body@Work (een samenwerking tussen het VU medisch centrum en TNO Kwaliteit van Leven). www.vitaalinpraktijk.nl

colofon

TEKSTEN
Jantien van Berkel
EINDREDACTIE
Arboriginals, Amsterdam
ONTWERP
Meester Ontwerpers, Amsterdam
DRUK
Drukwerkfabriek, Oosterhout

Appendix 2 Participant letter of invitation by HRM

Beste collega,

Bij deze wil ik u van harte uitnodigen om deel te nemen aan het onderzoek 'Vitaal in Praktijk' (VIP). Het doel van VIP bij het RIVM is het ontwikkelen en evalueren van een programma gericht op een gezonde leefstijl (bijv. meer bewegen en gezonde voeding) en het in stand houden van de mentale gezondheid (zoals bevlogenheid) bij RIVM medewerkers. Meer informatie over VIP treft u aan in de folder.

Via uw leidinggevende heeft u bijgevoegd pakket ontvangen. Wilt u de bijgevoegde antwoordkaart invullen en naar het onderzoeksteam terugsturen? U kunt hiervoor de antwoordenvelop gebruiken. U hoeft hier geen postzegel op te plakken.

Voor het onderzoek is het van groot belang dat er een groot aantal deelnemers is. Uw deelname wordt dus zeer gewaardeerd! Nadat u zich heeft aangemeld zal u worden uitgenodigd voor de eerste meting en de eerste vragenlijst ontvangen. Bij voldoende aanmeldingen zal het programma van start gaan. Na de start is aanmelding niet meer mogelijk. Deelname aan het programma kan tijdens de eerste 8 weken ongeveer anderhalf uur per week van uw tijd innemen. Na de eerste 8 weken kost deelname aan het programma u ongeveer een kwartier per week. Het programma duurt in totaal een half jaar. Er zijn 3 meetmomenten: vooraf, na 6 maanden en dan nog eens na 6 maanden. Een meetmoment bestaat uit een fysieke meting en een vragenlijst. Tussentijds krijgt u nog een kleine vragenlijst.

Wanneer u nog vragen hebt naar aanleiding van deze uitnodiging, kunt u contact opnemen met het VIP projectteam per email via: vipinonderzoek@vitaalinpraktijk.nl of telefonisch via 06- 22002759. Tevens is er een onafhankelijke arts, de heer Gallee, betrokken bij dit onderzoek.

Bij voorbaat hartelijk dank voor uw medewerking,
mede namens het VIP projectteam,

Met vriendelijke groet,

Nicole van Overbeek

Appendix 3: Participant registration form/ Informed consent

Antwoordkaart

Tekst:

Ja , ik doe mee aan VIP in Onderzoek!
Hierbij verstrek ik mijn contactgegevens, zodat iemand uit het projectteam contact met mij op kan nemen.

Met mijn deelname geef ik de onderzoekers toestemming mijn gegevens te verzamelen voor VIP in onderzoek. Gegeven worden verzameld door middel van een fysieke meting en 3 grote vragenlijsten (de eerste op zeer korte termijn, de tweede over een half jaar en de laatste over een jaar) en 2 kleine tussentijdse vragenlijsten. Tevens geef ik toestemming voor het opvragen van mijn ziekteverzuimgegevens bij de afdeling HRM en gebruik hiervan voor het onderzoek.

Mijn gegevens zullen niet aan derden (zoals collega's of leidinggevenden) worden verstrekt. Contactgegevens zullen alleen voor contact omtrent het onderzoek worden gebruikt. De onderzoeksgegevens worden los van de contactgegevens bewaard en zijn niet aan elkaar gekoppeld. De onderzoekers garanderen volledige geheimhouding. Rapportage zal alleen plaatsvinden op groepsniveau en nooit op individueel niveau, zodat gegevens niet naar mij te herleiden zijn.

Naam:
Telefoonnummer (werk):
Emailadres (werk):
Adres (werk):
Afdeling:
Fulltime/Partime
Werkdagen: ma/di/wo/do/vr
Werktijden: van __u tot ___u
Datum:
Handtekening:

Appendix 4: Supervisors accompanying letter

Beste leidinggevende,

Tijdens het MT overleg bent u geïnformeerd over het Vitaal in Praktijk (VIP) project bij het RIVM, een project waarbij een programma wordt ontwikkeld en geëvalueerd ter bevordering van een gezonde leefstijl (en voeding en bewegen in het bijzonder) en bevlogenheid van de RIVM medewerkers. Het VIP project wordt uitgevoerd door Body@Work, een samenwerking tussen TNO Kwaliteit van Leven en VU medisch centrum.

Het programma is in nauwe samenwerking met RIVM medewerkers ontwikkeld. In een eerste stap hebben de onderzoekers een aantal sleutelfiguren geïnterviewd. De tweede stap was het uitzetten van een korte vragenlijst, waarvoor u ook bent benaderd om werknemers te vragen. Vervolgens zijn 6 focusgroep interviews gehouden onder een willekeurige selectie van RIVM medewerkers. De programmaopzet is vervolgens geëvalueerd in 2 groepsdiscussies met medewerkers en sleutelfiguren. Dit alles heeft geleid tot een maat gemaakt programma voor het RIVM. Dit programma kan nu getoetst worden.

Hierbij treft u een aantal pakketjes aan. In die pakketjes zit een uitnodiging, tezamen met een informatiefolder over VIP. Tevens is een antwoordkaart met antwoordenvelop toegevoegd. Wilt u deze pakketjes aan uw medewerkers geven, met de vraag of zij zouden willen deelnemen?

Voor het onderzoek is het van groot belang dat er voldoende deelnemers zijn. Uw hulp hierbij wordt zeer gewaardeerd!

Wanneer u nog vragen hebt naar aanleiding van deze brief, kunt u contact opnemen met het projectteam van VIP via email: vipinonderzoek@vitaalinpraktijk.nl of telefonisch via: 06-22002759.

Bij voorbaat hartelijk dank voor uw medewerking,

Met vriendelijke groet,

Nicole van Overbeek

Appendix 5: Costs diary

Kostendagboekje

Deelnemerscode: ________________

Datum: ________________________

Kostendagboekje

Contactpersoon:
Jantien van Berkel
Van der Boechorststraat 7
1081 BT Amsterdam
vipinonderzoek@vitaalinpraktijk.nl
06- 22002759

Gebruik van zorg

1. Bent u in de afgelopen 6 maanden naar een zorgverlener geweest?

- ☐ Nee
- ☐ Ja, naar een diëtist(e) — Aantal bezoeken: ☐☐
- ☐ Ja, naar een (bedrijfs -) fysiotherapeut — Aantal bezoeken: ☐☐
- ☐ Ja, naar een Mensendieck/ Cesartherapeut — Aantal bezoeken: ☐☐
- ☐ Ja, naar een sportarts of sportmedisch adviescentrum — Aantal bezoeken: ☐☐
- ☐ Ja, naar een cardioloog — Aantal bezoeken: ☐☐
- ☐ Ja, naar een internist — Aantal bezoeken: ☐☐
- ☐ Ja, naar een psycholoog — Aantal bezoeken: ☐☐
- ☐ Ja, naar de huisarts — Aantal bezoeken: ☐☐
- ☐ Ja, naar de bedrijfsarts of bedrijfsmaatschappelijk werker of bedrijfsverpleegkundige — Aantal bezoeken: ☐☐
- ☐ Ja, naar een andere zorgverlener, namelijk:………………………..
…………………………………………………………………… Aantal bezoeken: ☐☐

2. Bent u in de afgelopen 6 maanden opgenomen geweest in het ziekenhuis?

- ☐ Nee
- ☐ Ja, aantal dagen: ☐☐☐

Appendix 6: Questionnaire
